# Management of suspicious nodules in lung cancer screening - a narrative review of monitoring strategies based on nodule features and further needs

**DOI:** 10.3389/fonc.2026.1744385

**Published:** 2026-06-03

**Authors:** Rodica Anghel, Vlad-Luca Moga, Antonia-Ruxandra Folea, Anca-Florina Zgură, Luiza-Georgia Şerbănescu, Radu-Valeriu Toma, Şerban-Andrei Marinescu, Andreea-Iren Şerban, Liviu Bîlteanu

**Affiliations:** 1 Faculty of General Medicine, Carol Davila University of Medicine and Pharmacy, Bucharest, Romania; 2 Oncological Institute Alexandru Trestioreanu, Bucharest, Romania; 3 Faculty of Biology, University of Bucharest, Bucharest, Romania; 4 Department of Preclinical Sciences, Faculty of Veterinary Medicine, University of Agronomic Sciences and Veterinary Medicine, Bucharest, Romania; 5 Laboratory for Molecular Nanotechnologies, National Institute for Research and Development in Microtechnologies - IMT Bucharest, Voluntari, Romania

**Keywords:** artificial intelligence, digital tools, LDCT screening, lung cancer, lung nodule, overdiagnosis, volume doubling time

## Abstract

**Background/objectives:**

Low-dose computed tomography screening plays a pivotal role in early lung cancer detection. This narrative review aims to evaluate nodule characteristics, especially volume doubling time (VDT), and their relevance to lung cancer suspicion, staging, and clinical outcomes, to support more accurate risk stratification in screening programs for lung cancer.

**Materials and methods:**

A literature search was conducted in PubMed, Scopus, and Web of Science, covering studies published from January 2012 to August 2024. A total of 27 studies (23 original and 4 reviews) were included. Key nodule features (VDT, size, attenuation, margins, histology, and stage) were extracted, reclassified, and analyzed to ensure standardized graphical comparison. Diagnostic performance metrics such as sensitivity, specificity, and predictive values of VDT were also assessed.

**Results:**

Findings revealed that all malignant nodules had a VDT under 200 days, with the shortest VDTs observed in aggressive histological subtypes and advanced disease stages. PET-CT positivity correlated with shorter VDTs, and never-smokers exhibited faster nodule growth than ever-smokers. Stage-specific growth patterns showed a trend of decreasing VDT with disease progression. However, variability in study designs and classification criteria made it necessary to implement standardization. Digital tools in the field of lung imaging may be valuable assets in early detection and risk prediction and could minimize inter-reader variability and thus overdiagnosis.

**Conclusions:**

VDT is a valuable indicator for assessing nodule malignancy risk but should be integrated into multifactorial risk models. Standardizing VDT reporting and incorporating it into personalized lung cancer screening algorithms could enhance early detection and reduce overtreatment. Digital tools can support this integration by enabling accurate, automated VDT calculations, improving measurement consistency, and facilitating the incorporation of volumetric and attenuation data into advanced risk prediction models, provided that healthcare professionals receive proper training to use these tools effectively.

## 1 Introduction

Lung cancer (LC) remains one of the leading causes of cancer-related mortality worldwide (1) prompting numerous clinical trials (such as the National Lung Screening Trial from the United States or the Dutch-Belgian Randomized Lung Cancer Screening trial) investigating low-dose computed tomography (LDCT) screening as a strategy to improve prognosis (2, 3). LDCT screening has been widely recognized as an effective method for reducing mortality and facilitating early LC detection (2, 4). However, lung cancer screening (LCS) is a long-term, ongoing process rather than a one-time intervention, as a significant number of indeterminate pulmonary nodules may necessitate diagnostic evaluations (such as surgical, percutaneous, or bronchoscopic biopsies) (5), follow-up imaging, or annual repeat screenings (6–8).

LC is often diagnosed at a metastatic stage, resulting in a low 5-year survival rate of 10–15% (9, 10). While several studies report that the 5-year survival rate for Stage I LC exceeds 80%, this rate drops to near 0% for Stage IV disease (11, 12). However, other studies have indicated different survival outcomes, with Stage IA at 61% and Stage IB at 38% (10), which still highlight the critical importance of early detection. The primary goal of LCS is to identify the disease at a curable stage.

At the European level, there is no unified LCS program; within the member states, pilot programs are being organized with some success at the local or regional level. Recent initiatives have been developed in the past few years to facilitate the implementation of lung cancer screening programs across Europe. SOLACE project (https://europeanlung.org/solace/), an innovative EU4Health initiative under Europe’s Beating Cancer Plan, is aiming to design flexible LCS methodology primarily using LDCT as a simple and effective method for detecting pulmonary nodules. The current lack of a unified European approach to lung cancer screening demonstrates the urgent need for standardized, evidence-based diagnostic criteria that can be uniformly applied across diverse healthcare settings. Because regional pilot programs currently rely on variable follow-up recommendations, establishing a clear, comprehensive picture of nodule features - such as volume doubling time (VDT) and imaging characteristics - is essential for harmonizing these fragmented screening efforts. By identifying converging criteria for malignancy, this study aims to support broad initiatives like SOLACE in designing flexible, yet standardized, screening methodologies that effectively reduce overdiagnosis and improve screening efficiency across Europe

Overdiagnosis is a potential limitation of LCS, occurring when a detected cancer would not have become symptomatic during the patient’s lifetime or contributed to mortality (13). The high frequency of early-stage cancer detection raises concerns about overdiagnosis, as some cancers may never progress to clinical disease and would remain undiagnosed without screening. This phenomenon carries significant risks, including unnecessary medical interventions, additional diagnostic procedures, and overtreatment, ultimately leading to an avoidable burden on patients (14). Quantitative estimates of overdiagnosis vary widely depending on the length of follow-up and nodule histology. In the initial analysis of the National Lung Screening Trial (NLST), the calculated upper bounds probability of overdiagnosis was 18.5% for all lung cancers, but it was notably higher (78.9%) for indolent histologic subtypes such as bronchoalveolar carcinomas (15). However, extended long-term follow-up of the NLST cohort (median 12.3 years) revealed that the actual overdiagnosis rate was only 3% (16). Similarly, the Dutch-Belgian NELSON trial demonstrated that overdiagnosis rates dropped from 19.7% at 10 years to just 8.9% after 11 years of follow-up (16). Comparative modeling studies also estimate that overdiagnosis accounts for approximately 8.7% to 13.5% of all screen-detected lung cancers (17). Furthermore, utilizing quantitative imaging markers like VDT can help identify these overdiagnosed cases; studies suggest that only about 5% to 25.8% of screening-detected cancers exhibit the slow-growing or indolent behavior (VDT > 400 days) characteristic of overdiagnosis (18).

Current follow-up recommendations are primarily based on nodule diameter and solidity (19). However, research suggests that these criteria alone are insufficient to fully assess nodule complexity, and visual interpretations of solidity are susceptible to inter-rater variability (20). VDT was also utilized as a tool to identify slow-growing or indolent cancers, which may be subject to overdiagnosis, and to compare their clinical outcomes with those of faster-growing tumors (18).

The objective of this study is to provide a comprehensive picture, within LCS, of nodule features (VDT, dimension, imagistic features) and their associations with LC positive diagnosis and disease features. In other words, this study aimed to analyze features that could facilitate the differentiation between suspicious and benign nodules, thereby reducing overdiagnosis and improving screening efficiency. This approach aims to contribute to the reduction of overdiagnosis and minimize unnecessary invasive interventions for patients. We believe this can be achieved by integrating digital tools into the imaging workflow, with AI applications and radiomics-driven extraction of quantitative parameters from computed tomography (CT) images having the potential to increase diagnostic accuracy and enable personalized screening strategies based on imaging biomarkers. This, of course, can only be attained through digital training programs for medical personnel, not only for radiologists but also for nurses and radiology technicians.

The remainder of this article is organized as follows: Section 2 (Materials and Methods) outlines the methodological framework of this narrative review, detailing the systematic the literature search, data extraction, and the reclassification approach used to ensure standardized graphical representation of both quantitative and qualitative nodule variables. Section 3 (Results) presents a comparative analysis of key nodule characteristics across the included studies, focusing on VDT, size, attenuation, margins, and staging. Section 4 (Discussion) explores the clinical significance of VDT as an indicator of tumor aggressiveness and evaluates its integration into comprehensive risk stratification models, supported by digital tools. Finally, Section 5 (Conclusions) summarizes the rationale for incorporating standardized VDT thresholds and automated digital applications into personalized diagnostic algorithms to improve early lung cancer detection and optimize patient outcomes.

## 2 Materials and methods

### 2.1 Literature search

While this study is designed as a narrative review, a systematic literature search approach was employed to ensure a rigorous, unbiased, and reproducible selection of the evidence.

#### 2.1.1 Database search strategy

A comprehensive literature search was performed in August 2024 using the Scopus, PubMed, and Web of Science databases, covering studies published between January 2012 and August 2024. The search formula employed the following query to filter relevant articles and abstracts; the search was conducted within the title and abstract fields:

(lung OR pulmonary) AND cancer AND screening AND nodule.

#### 2.1.2 Inclusion and exclusion criteria

To explicitly define the inclusion and exclusion criteria for final article selection, we established strict parameters to filter the initial search results. Inclusion criteria required that the studies be (1) original research articles or comprehensive reviews (2); published in the English language (3); published between January 2012 and August 2024; and (4) studies that presented reliable, quantitative, or comprehensive characterizations of suspicious pulmonary nodules (e.g., distributions by size, VDT, qualitative imagistic features, or associated disease staging) within the context of LDCT in LCS. Conversely, the exclusion criteria were (1) non-research publications, including letters, short communications, and meeting abstracts (2); records published in languages other than English; and (3) studies that lacked relevant extractable data regarding the specific quantitative or qualitative nodule variables targeted by this review.

This curated selection intentionally prioritized study quality and data completeness over quantity. This enabled the standardized mathematical comparison of continuous variables (e.g., exact VDT and nodule dimensions) across large, pooled cohorts, and facilitated the systematic evaluation and harmonization of categorical features (e.g., nodule attenuation, margins, and histopathological staging). Expanding the scope to include additional studies with less rigorous or incomplete reporting would have introduced significant heterogeneity, thereby compromising the depth, reliability, and direct comparability of this analysis.

The significant reduction from 5,399 initial records to 27 final studies reflects the scarcity of literature providing the granular, standardized nodule metrics required for this specific analysis. While a broad search query was necessary to ensure no relevant literature was missed, the vast majority of initial hits were excluded because they either did not focus specifically on LDCT screening, or they lacked the robust quantitative data (such as exact VDT or dimensions) and comprehensive qualitative characterizations necessary for our comparative framework. However, we acknowledge that applying such stringent criteria introduces a degree of selection bias into this review, as studies with highly complete data reporting often involve specifically selected patient cohorts.

#### 2.1.3 Screening workflow

**Figure 1:**
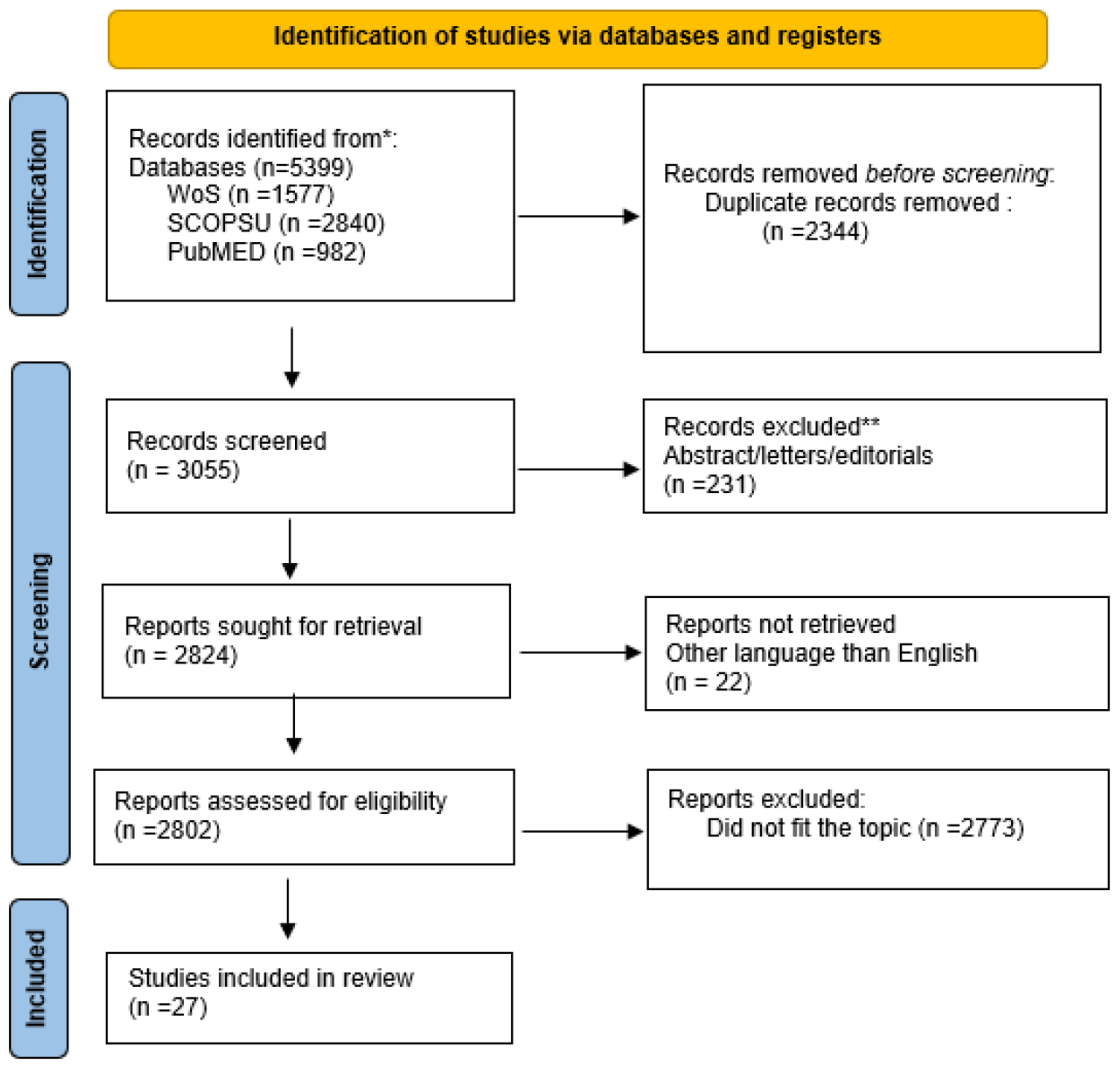
Flow-chart showing the selection processes.

The selection process follows a PRISMA-compliant screening workflow, which is presented in summary by the flowchart in **Figure 1**. Initially, 5,399 records were identified across the three databases. After eliminating 2,344 duplicate records, an additional 231 records were excluded because they were not reviews or research articles (letters, short communications, meeting abstracts, and other non-research articles), while an additional 22 records were removed due to being published in languages other than English. This resulted in 2,802 records assessed for eligibility. Following further screening, 2,773 records were excluded because they did not align with our objective to find extensive reliable studies presenting comprehensive or quantitative characterization of suspicious pulmonary nodules within LCS, leaving 25 research articles and 4 reviews, which were included in the final analysis. **Table 1**, presents the study design of the selected original research articles listed in chronological order of their publication.

**Table 1:** The design of the 25 research studies included in this analysis.

Study	Time directionalities	Experimental control	Randomization
Saghir et al. (2012) (14)	Prospective	Interventional	Randomized
Veronesi et al. (2012) (18)	Retrospective	Observational	Nonrandomized
Horeweg et al. (2013) (21)	Prospective	Interventional	Randomized
Nahorecki et al. (2014) (42)	Prospective	Interventional	Randomized
Pinsky et al. (2014) (23)	Prospective	Observational	Nonrandomized
Zhao et al. (2014) (24)	Retrospective	Observational	Nonrandomized
Horeweg et al. (2014) (22)	Retrospective	Observational	Nonrandomized
McKee et al. (2016) (25)	Retrospective	Observational	Nonrandomized
Walter et al. (2016) (26)	Retrospective	Observational	Nonrandomized
Kavanagh et al. (2018) (27)	Prospective	Observational	Nonrandomized
Walter et al. (2019) (1)	Retrospective	Observational	Nonrandomized
Hammer et al. (2019) (28)	Retrospective	Observational	Nonrandomized
Tang et al. (2019) (29)	Retrospective	Observational	Nonrandomized
Kim et al. (2020) (30)	Retrospective	Observational	Nonrandomized
Kim et al. (2021) (31)	Retrospective	Observational	Nonrandomized
Wang et al. (2021) (32)	Prospective	Interventional	Nonrandomized
Burks et al. (2021) (15)	Prospective	Observational	Nonrandomized
Andersson et al. (2021) (33)	Prospective	Observational	Randomized
Manjunath et al. (2022) (34)	Prospective	Interventional	Randomized
Akpoviroro et al. (2022) (44)	Retrospective	Observational	Nonrandomized
Auger et al. (2023) (35)	Prospective	Observational	Nonrandomized
El Alam et al. (2023) (36)	Retrospective	Observational	Nonrandomized
Lin et al. (2023) (37)	Retrospective	Observational	Nonrandomized
Gulati et al. (2024) (38)	Retrospective	Observational	Nonrandomized
Tang et al. (2024) (39)	Retrospective	Observational	Nonrandomized

To clarify the methodological scope of this review, it is important to note that the discussion regarding radiomics and AI herein is conceptual rather than applied. Our study design relies entirely on the systematic extraction, reclassification, and synthesis of published clinical and conventional radiological data (e.g., VDT, nodule size, attenuation) from existing literature. We did not perform primary computational extraction of radiomic features or apply novel AI/deep learning algorithms to raw computed tomography datasets. Instead, we conceptually explore how these advanced digital tools are increasingly utilized in the literature to automate volumetric assessments, minimize inter-reader variability, and optimize lung cancer risk prediction models (12, 32).

While the final selection yielded 27 articles (23 original research studies and 4 reviews), this focused cohort was intentionally established to allow for a highly detailed qualitative and quantitative investigation. The strict selection criteria prioritized study quality and data completeness over mere quantity, ensuring that each included article provided robust, extractable data. Quantitatively, this curated selection enabled the standardized mathematical comparison of continuous variables - such as exact VDT and nodule dimensions - across large, pooled cohorts. Qualitatively, it facilitated the systematic evaluation and harmonization of categorical features, including nodule attenuation, margins, and histopathological staging. Expanding the scope to include additional studies with less rigorous or incomplete reporting would have introduced significant heterogeneity, thereby compromising the depth, reliability, and direct comparability of this analysis.

#### 2.1.4 Quality and risk of bias assessment

To evaluate the methodological rigor and potential risk of bias of the original research studies included in this review, a formal assessment was conducted using the QUADAS-3 (Quality Assessment of Diagnostic Accuracy Studies, version 1.2) tool (40). This tool evaluates studies across four key domains (1): Participants (2), Index Test (3), Target Condition, and (4) Analysis. For each study, these domains were systematically assessed using predefined signaling questions to determine the risk of bias and concerns regarding applicability to the review’s synthesis questions. An overall judgment was subsequently assigned to each study, classifying the overall risk of bias and applicability concerns as “Low,” “High,” or “Insufficient Information” based on the domain-level ratings.

### 2.2 Nodule variables

The analysis focused on both qualitative characteristics of nodules (referred to as variables throughout the article) and especially on quantitative characteristics, with the aim of identifying converging criteria indicative of malignancy. To support this objective, data related to VDT, growth pattern, size, attenuation, margins, location, histology, and staging frequently reported across multiple studies were collected and formed the basis of the analysis. The selected groups were examined, and percentages were calculated for each defined category. These categories were carefully structured to ensure comparability across studies, enabling clear visualization and meaningful comparison.

Quantitative data were selected for comparative analysis between different studies, such as mean and median values for VDT and size, while qualitative data included size, margins, stage, attenuation, histopathological type, and nodule location. To standardize statistical terminology across all comparisons, quantitative variables (such as VDT and nodule size) are consistently reported as either means with standard deviations (mean ± SD) or medians with interquartile ranges (median [IQR]) or ranges, strictly adhering to their original reporting. Proportions are presented as percentages (%) with 95% confidence intervals (CI) where applicable.

### 2.3 Data processing and visualization

Categorical data were regrouped and reclassified to ensure homogeneous graphical representation and direct comparability across the selected studies. The primary rationale for this reclassification was the significant inter-study heterogeneity in nodule reporting and management protocols. For example, some cohorts (such as the NELSON trial) relied heavily on volumetric assessments and specific VDT thresholds, whereas others utilized linear diameter measurements guided by systems like Lung CT Screening Reporting and Data System (Lung-RADS) (22, 25). To overcome these methodological disparities, we justified the harmonization of the extracted data into standardized, overarching categories. For instance, in the VDT category, we selected a time interval of 0 to 1400 days to accommodate data from all authors. Similarly, for the LC stage category, patients were reclassified according to the AJCC staging system (Stages I-IV), as this was the only approach that allowed a comparative representation of values across multiple studies. These methodological choices are further detailed in Section 3: Results. For studies that presented data across multiple categories, these were consolidated under the “Other” category to maintain consistency. Additionally, some authors applied more restrictive cohort selection criteria, leading to the absence of certain categories in their datasets, even though these were considered in our analysis.

To visualize patient distribution across different categories, visualization methods were selected based on the nature of the variables to ensure optimal data synthesis. Line graphs were explicitly chosen to represent continuous or sequential data - such as nodule distribution over time (or VDT) or progressive size intervals - to highlight growth trends and continuous shifts in distributions across large, pooled datasets. Conversely, bar graphs were selected for discrete, categorical variables - such as histopathological type, nodule margins, disease stage, and attenuation. For these categorical variables, bar graphs (centered on the mean or median values and bounded by minimal and maximal reported values) were deemed most appropriate to facilitate clear, side-by-side comparative representations of frequencies and data ranges across the heterogeneous selected studies.

An attempt was made to graphically compare the Lung-RADS classification across the selected studies; however, this was not feasible, as the authors reported only specific categories or grouped multiple categories together (e.g., Lung-RADS categories 1 and 2 are grouped together). Consequently, the data could not be reclassified in a manner that would allow for a consistent and unified graphical representation.

## 3 Results

### 3.1 Risk of bias assessment

Based on the QUADAS-3 (version 1.2) assessment, the methodological quality and risk of bias across the 25 selected original estimates varied significantly. Overall, 10 of the 25 studies (40%) demonstrated a “Low” overall risk of bias, indicating that all four evaluated domains (Participants, Index Test, Target Condition, and Analysis) were rated as having a low risk of introducing bias. Conversely, 15 studies (60%) were judged to have a “High” overall risk of bias.

The high risk of bias across the literature (**Table 2**) was predominantly driven by issues in participant selection (Domain 1), where 36% of the studies were flagged as high risk due to retrospective, multi-gate (case-control), or highly restrictive cohort selection methods (e.g., exclusively analyzing surgically resected nodules). Differential or partial verification biases in confirming the target condition (Domain 3) also contributed to the high risk of bias in 24% of the studies, often because different reference standards were applied depending on the index test results or imaging findings.

**Table 2:** Summary of risk of bias across QUADAS-3 domains.

QUADAS-3 domain	Low risk of bias	High risk of bias	Insufficient information
Domain 1: Participants	64% (n=16)	36% (n=9)	0% (n=0)
Domain 2: Index test	96% (n=24)	4% (n=1)	0% (n=0)
Domain 3: Target condition	76% (n=19)	24% (n=6)	0% (n=0)
Domain 4: Analysis	80% (n=20)	20% (n=5)	0% (n=0)

The Index Test (Domain 2) and Analysis (Domain 4) domains were generally robust across the included literature. The index tests applied in the studies were overwhelmingly characterized by objective, pre-specified thresholds (96% low risk), and statistical analyses appropriately handled missing data and patient exclusions in 80% of the studies. None of the studies lacked sufficient information to make a risk of bias judgment.

### 3.2 Volume doubling-time

#### 3.2.1 Distribution of VDT across cohorts

**Figure 2:**
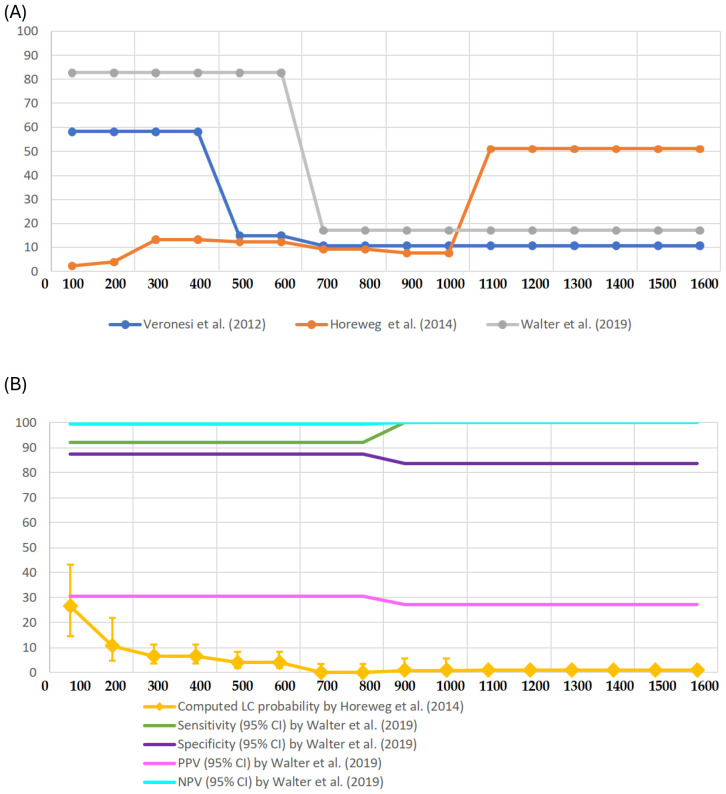
VDT as a marker for suspicious nodules in LCS. **(A)** Nodules absolute frequency (percentages) distribution on VDT. A time interval of 0–1600 days was selected for representing VDT and categorized the values from the reviewed studies accordingly. Intervals varied across the studies, so to enable the comparability the timescale axis has been divided in 100 days units. **(B)** Lung cancer probability (22) and the sensitivity, specificity, PPV, and NPV of VDT (1) as a diagnostic tool.

**Figure 2A** illustrates the distribution of nodules according to VDT, based on three studies (1, 18, 22). Patient distribution is expressed as relative frequency over a VDT interval of 0–1600 days, corresponding to the maximum values reported in the literature. Although the original studies used heterogeneous categorizations - such as 0-400, 400-600, 600–1000 days (18), <590 vs. >590 days (1), or 200-day increments (22) - all data were harmonized into 100-day intervals to ensure comparability.

Distinct growth patterns emerge across studies. Veronesi et al. (2012) (17) reported a predominance of nodules with VDT 100–400 days (58.3%), followed by a sharp decrease in the 400–600 days range and a small proportion of stable nodules (>600 days, 10.8%). Similarly, Walter et al. (2019) (1) observed a high proportion of nodules with short VDTs (82.84%), with fewer cases exceeding 600 days (17.16%). In contrast, Horeweg et al. (2014) (22) described a more gradual distribution, with fewer rapidly growing nodules and a substantial proportion of slow-growing lesions, reaching 51.07% for VDT >1000 days. A VDT threshold of 400 days has been proposed to distinguish overdiagnosed cases from true malignancies, with an estimated overdiagnosis rate of approximately 5% (41).

#### 3.2.2 Predictive value of VDT thresholds

**Figure 2B** presents the diagnostic performance of VDT as a malignancy indicator, comparing two categories (0–600 days and 600–1600 days). The figure integrates LC probability derived from prior estimates (22) with sensitivity, specificity, positive predictive value (PPV), and negative predictive value (NPV) reported elsewhere (1), allowing direct comparison across VDT ranges.

Shorter VDT values are associated with a higher probability of malignancy. Based on data from the NELSON trial, within the 0–600 day interval, LC probability ranges from a high of 26.5% for nodules with a VDT <100 days, steadily decreasing to 4.0% for those in the 400–600 day range (22). This indicates substantial variability in malignancy risk even within fast-growing categories. Diagnostic performance also differs significantly based on the thresholds applied. When utilizing a strict VDT cutoff of days, sensitivity is 92.0% and specificity is 87.4%, with a PPV of 30.7% and a NPV of 99.4% (1). While this reflects a high detection capacity, the low PPV indicates a considerable rate of false positives. Conversely, relying solely on a slower VDT (>590 days) yields an NPV of 99.4% but misses a small fraction of malignancies. However, diagnostic accuracy can reach near-complete discrimination when combining growth metrics with volume limits. By classifying a nodule as positive if it exhibits either a VDT days OR a volume mm³, sensitivity and NPV both reach 100.0%, while specificity remains robust at 83.7%, thereby minimizing the misclassification of true malignancies (1).

Diagnostic performance differs between categories. For VDT 0–600 days, sensitivity is 92% and specificity is 87.4%, with PPV of 30.7% and NPV of 99.4%. This reflects high detection capacity but a considerable rate of false positives. In contrast, for VDT 600–1600 days, sensitivity and NPV reach 100%, while specificity increases to 99.4%, indicating near-complete discrimination and minimal misclassification (1).

### 3.3 Associations with volume doubling time

#### 3.3.1 Influence of smoking status on nodule growth

**Figure 3:**
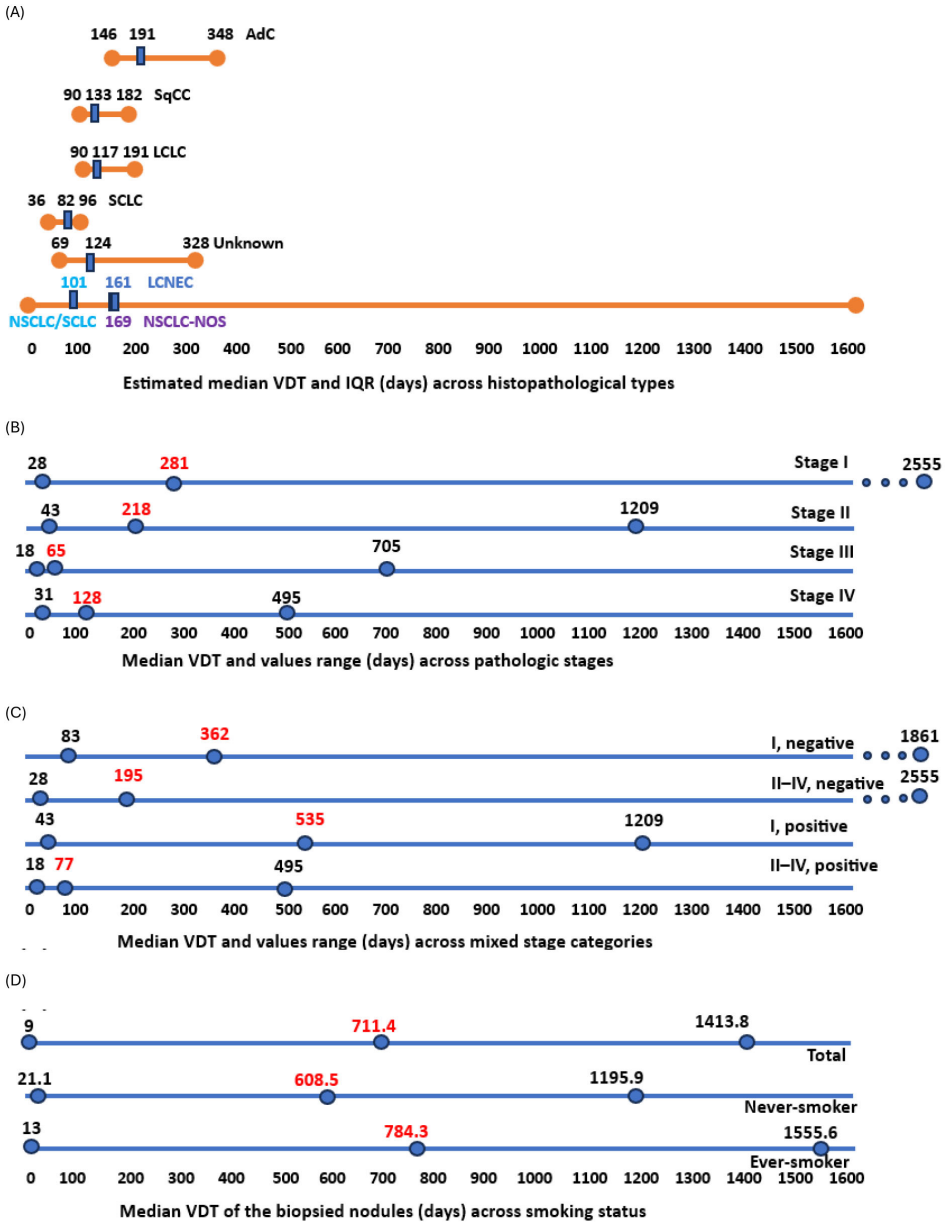
VDT as a LCS marker for LC suspicious nodules: **(A)** Median VDT and IQR across the histopathological phenotypes according to (26). Median and IQR was not provide for adenosquamous lung carcinoma (AdSqLC), while for large cell neuroendocrine carcinoma (LCNEC), non-small cell lung cancer/small cell lung cancer (NSCLC/SCLC), and non-small cell lung cancer – not otherwise specified (NSCLC-NOS), the standard deviation was not provided so IQR was not available. **(B)** Median VDT values and their ranges across various LC stages (17). Dotted lines were used when the interval was too large to be fully displayed on the bar. **(C)** Median VDT values and their range across different classes combining LC stages and PET-CT results (17). Dotted lines usage is similar to **(B)**. **(D)** Median VDT values and their range across a cohort of subjects exhibiting suspicious pulmonary nodules which is then divided into never-smokers and ever-smokers (30).

**Figure 3** summarizes the relationship between VDT and key clinicopathological factors, including histology, tumor stage, PET-CT findings, and smoking status. **Figure 3A** presents median VDT and IQR across histopathological subtypes. Some data were incomplete, with missing median/IQR for adenosquamous carcinoma (AdSqLC) and missing IQR for large cell neuroendocrine carcinoma (LCNEC), non-small cell lung cancer/small cell lung cancer (NSCLC/SCLC), and non-small cell lung cancer – not otherwise specified (NSCLC-NOS) (20). The shortest VDTs (~100 days) were observed in aggressive subtypes such as large cell lung carcinoma (LCLC), small cell lung carcinoma (SCLC), and non-small cell lung carcinoma/small cell lung carcinoma (NSCLC/SCLC) (26).

**Figure 3B** illustrates VDT distribution by stage. Early stages (I–II) show wide variability, with median VDTs exceeding 200 days (18), whereas advanced stages (III–IV) exhibit shorter and more homogeneous VDTs, reflecting faster tumor growth (Stage III: median 65 days, range 18–705; Stage IV: median 128 days, range 31–495). **Figure 3C** further stratifies VDT by PET-CT status. In Stage I, PET-negative nodules have a longer median VDT (362 days) compared to PET-positive cases (195 days). A similar pattern is observed in Stages II–IV, with markedly longer VDT in PET-negative cases (535 days) versus PET-positive nodules (77 days) (18).

**Figure 3D** compares VDT according to smoking status in a cohort of 37,436 subjects (30). Never-smokers show a shorter mean VDT (608.5 ± 587.4 days) than ever-smokers (mean 784.3 ± 771.3 days), indicating faster growth but with lower variability. The overall mean VDT is 711.4 ± 702.4 days, consistent with generally slow nodule progression.

#### 3.3.2 VDT correlation with disease stage and PET-CT positivity

**Figure 4:**
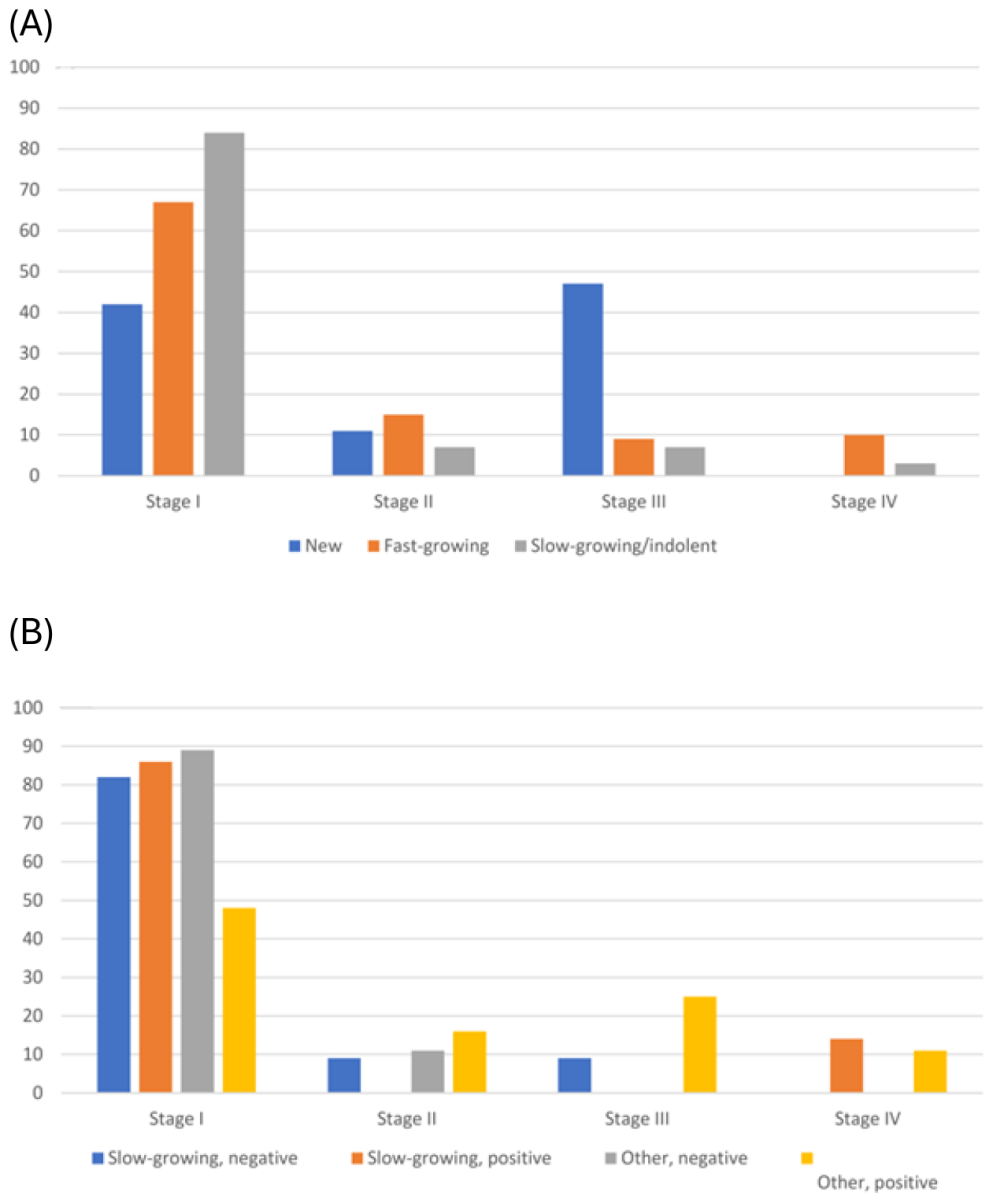
Nodule distribution by disease stage based on growth pattern: Veronesi et al. (2012) defined Fast-growing nodules as those with a VDT (Volume Doubling Time) of less than 400 days, Slow-growing nodules as those with a VDT between 400 and 600 days, and Indolent nodules as those with a VDT ≥ 600 days. **(A)** A visual representation in percentages was created to illustrate the distribution of nodules by disease stage and growth status (new, fast-growing, or slow-growing/indolent), based on data from Veronesi et al. (2012) (18). **(B)** The same author (18) also categorized nodules based on PET-CT results (Slow-growing, negative and positive and Other negative and positive). These values were graphically represented as percentages, offering a visualization of the proportion of nodules classified based on PET-CT findings.

**Figure 4A**, adapted from a single-center cohort study (18), illustrates the LC stages distribution according to nodule growth patterns (new, fast-growing, and indolent). Stage I predominates across all categories, accounting for 84% of indolent, 67% of fast-growing, and 42% of new nodules. Stage II is less frequent, with similar proportions for new and fast-growing nodules (11–15%) and fewer indolent cases (7%). Stage III is mainly associated with new nodules (47%), while fast-growing (9%) and indolent nodules (7%) remain uncommon. Stage IV is the least represented, with few fast-growing (10%) and indolent nodules (3%) and no new nodules observed.

**Figure 4B** further stratifies these patterns by combining the LC stage with PET-CT status (18). In Stage I, slow-growing nodules - both PET-negative and PET-positive - predominate (>80%), while PET-negative new and fast-growing nodules (“other, negative”) are similarly frequent (89%). In contrast, PET-positive new/fast-growing nodules (“other, positive”) are less common (48%). In Stage II, all categories decrease, with slow-growing negative and “other, negative” nodules ranging from 9–11%, while “other, positive” nodules increase slightly (16%); no slow-growing PET-positive nodules are observed. In Stage III, “other, positive” nodules become predominant (25%), whereas slow-growing PET-negative nodules remain low (9%), and other categories are absent. In Stage IV, slow-growing PET-positive nodules persist (14%) but are less frequent than in Stage I, while “other, positive” nodules decrease (11%), and slow-growing PET-negative nodules are no longer observed.

### 3.4 Nodule size

While dynamic growth kinetics (VDT) and metabolic activity (PET-CT) provide vital functional insights into tumor behavior, physical dimensions remain the most fundamental and universally applied baseline metric in lung cancer screening. Nodule size not only dictates the initial clinical risk categorization but also serves as the primary structural anchor against which all future volumetric changes are measured.

#### 3.4.1 Nodule diameter and volume distributions

**Figure 5:**
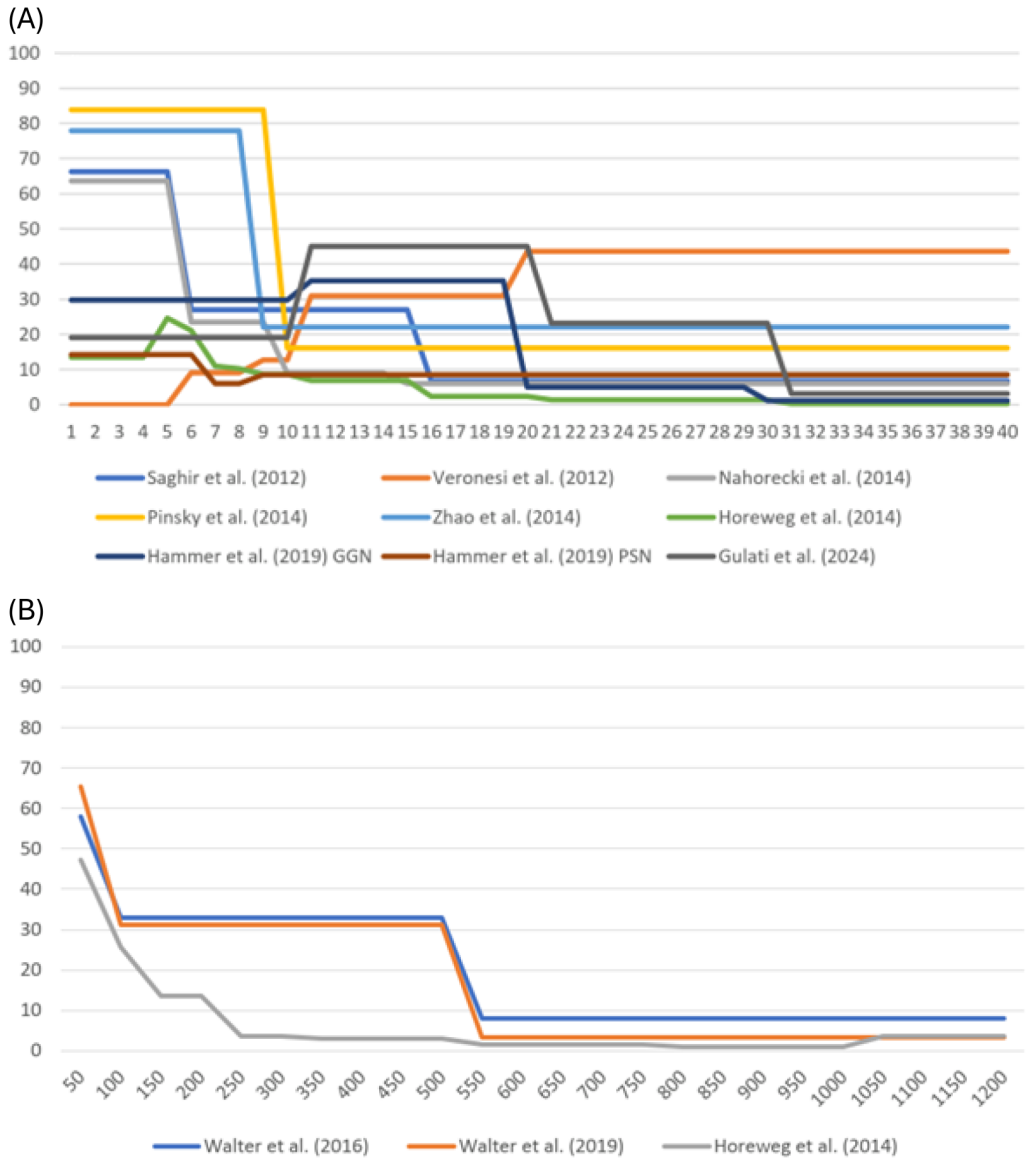
Distribution of nodules based on CT measurements: **(A)** distribution of nodules’ largest dimension based on different studies, respectively (14, 18, 22–24, 28, 38, 42). **(B)** distribution of nodules volumes using data extracted from three studies, respectively (18, 22, 24).

**Figure 5A** summarizes nodule diameter distribution across eight studies (14, 18, 22–24, 28, 38, 42), standardized to a 0–40 mm scale. Smaller nodules (0–10 mm) predominate in Saghir et al. (2012), Pinsky et al. (2024), and Hammer et al. (2019) (14, 23, 28), whereas mid-sized nodules (10–25 mm) are more evenly distributed in Veronesi et al. (2012), Zhao et al. (2014), and Horeweg et al. (2014) (18, 22, 24). Larger nodules (25–40 mm) are more frequent in Nahorecki et al. (2014), Hammer et al. (2019) (part solid nodules, PSN), and Gulati et al. (2024) (28, 38, 42).

**Figure 5B** presents nodule volume distribution from three studies (1, 22), using a 0–1400 mm³ scale. Small nodules (0–200 mm³) are predominant, with a peak in the 0–100 mm³ range (~70%). Walter et al. (2019) report the highest proportion (65.42%), followed by Walter et al. (2016) and Horeweg et al. (2014) (58% and 47.27%, respectively) (1, 22). Proportions decrease progressively between 200 and 600 mm³ and remain low (<10%) above 600 mm³, suggesting fewer large nodules or their preferential classification as malignant.

**Figure 6:**
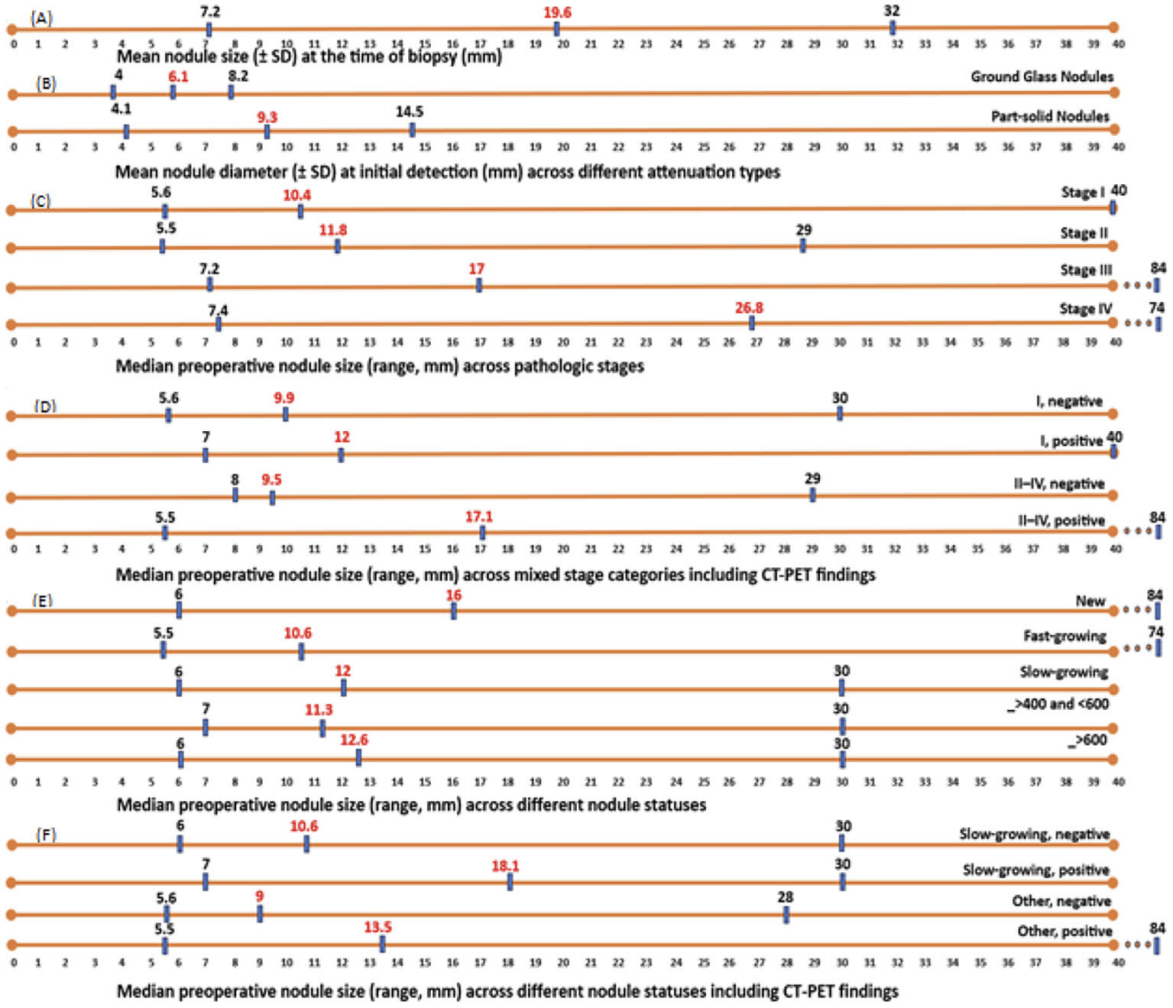
Nodule size across various categories. **(A)** Mean nodule size along with standard deviation at the time of biopsy in mm (30). **(B)** Mean nodule diameter (± SD) at initial detection in mm across different attenuation types: Ground-glass Nodules and Part-solid nodules (31). **(C)** Median preoperative nodule size and their ranges in mm across LC pathological stages (18). Dotted lines were used when the interval was too large to be fully displayed on the bar. **(D)** Median preoperative nodule size and their ranges in mm across mixed stage categories including PET-CT findings (18). Dotted lines usage is similar to **(C)**. **(E)** Median preoperative nodule size and their ranges in mm across different nodule statuses (18). Dotted lines usage is similar to **(C)**. **(F)** Median preoperative nodule size and their ranges in mm across different nodule statuses including PET-CT findings (18). Dotted lines usage is similar to **(C)**.

**Figure 6A-F** present diameter statistics (0–40 mm), consistently reported as mean ± SD or median [IQR] or range, depending on the available data from the original studies. For example, the mean diameter at biopsy is 19.6 mm (30). At detection, smaller diameters are observed for ground-glass nodules (6.1 ± 2.1 mm) compared to part-solid nodules (9.3 ± 5.2 mm) (43).

#### 3.4.2 Associations of size with staging and growth kinetics

**Figure 6C** shows a progressive increase in median nodule diameter across pathological stages (I–IV), from 10.4 mm (Stage I) to 11.8 mm (Stage II), 17 mm (Stage III), and 26.8 mm (Stage IV) (17). **Figure 6D** further stratifies diameter by stage and PET-CT status. In Stage I, median diameters are 9.9 mm (PET-negative) and 12 mm (PET-positive), while in Stages II–IV they are 9.5 mm and 17.1 mm, respectively, indicating larger nodules in PET-positive cases.

**Figure 6E** presents median preoperative diameters based on growth patterns. New nodules are largest (16 mm), followed by slow-growing (12 mm) and fast-growing nodules (10.6 mm). Similarly, nodules with VDT >600 days are larger (12.6 mm) than those with VDT 400–600 days (11.3 mm) (18). **Figure 6F** combines growth pattern and PET-CT status, showing that slow-growing nodules (both PET-negative and PET-positive) have larger diameters (10.6 and 18.1 mm) compared to other growth categories (9 and 13.5 mm) (18).

### 3.5 Qualitative computed tomography features

While quantitative metrics such as VDT and diameter provide essential baseline risk stratification, they must be interpreted in conjunction with morphological characteristics to form a complete diagnostic picture. Integrating these dynamic growth parameters with qualitative imaging findings - specifically nodule attenuation and margin irregularity - provides a seamless transition into a more comprehensive radiological profile.

#### 3.5.1 Nodule attenuation

**Figure 7:**
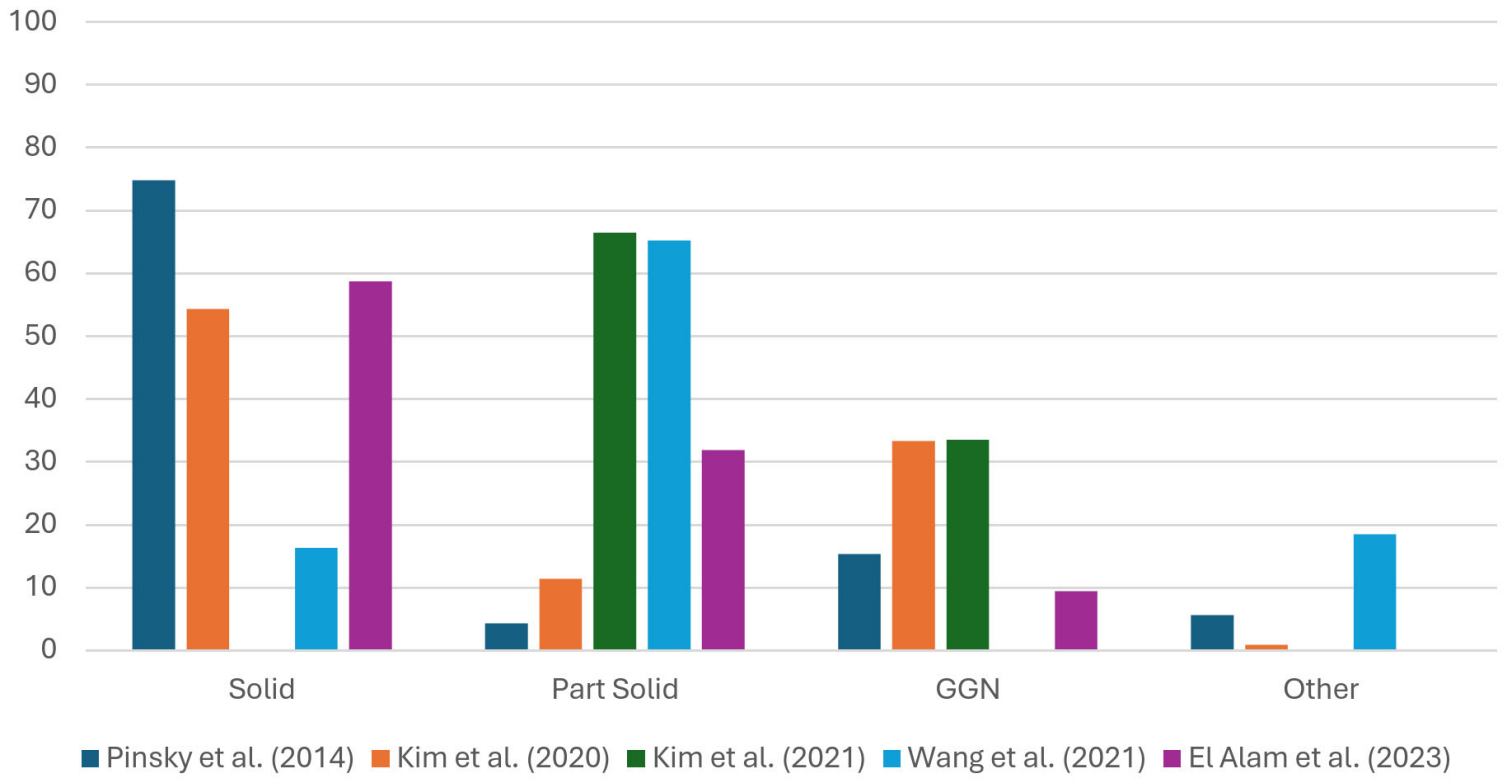
Distribution of pulmonary nodules based on attenuation categories (solid, part-solid, GGN, other) The nodules were categorized and visually represented in percentage across three primary classifications present in all studies, except for Kim et al. (2021) (31), who omitted the part-solid category, and Wang et al. (2021) (32), who did not include GGN (Ground-Glass Nodules). The primary categories analyzed were solid, part-solid, and GGN, while classifications introduced by different authors were grouped under a unified “Other” category. Additionally, Pinsky et al. (2014) (23) referred to solid nodules as “soft tissue.” Not all authors [Kim et al. (2021) (31), Wang el al (2021) (32). and El Alam et al. (2023) (36)] reported nodules within the “Other” category.

Nodule distribution across attention categories (23, 30–32, 36) are presented **Figure 7**. With only two exceptions in which the part-solid category (31) and the ground glass nodule (GGN) category were not included, all the other papers used the following categories: solid, part-solid, and GGN, while other classifications defined by different authors were consolidated into a single category: “Other.” Some authors (23) referred to solid nodules as “soft tissue” and some others (31, 32, 36) did not report pathological entities that can be included in the “Other” category. There are differences between studies, illustrated, for instance, by differences in the reported proportions of higher proportions of part-solid nodules in some studies [i.e., 66.5% (31) and 65.2% (32)] than the reported values in the other ones [i.e., 31.8% (36) or even a very small percentage, 4.3% (23)]. There is also variability in the proportions of solid nodules, with the highest reported value being 74.8% (23), while other authors report lower values, such as 58.7% (36) or 54.3% (30) or even 16.3% (32), which is the lowest percentage found in the included studies. For GGN, two studies report similar proportions [33.3% (30) and 33.5% (31)], with significantly reduced values but different from each other being reported otherwise (15.3% (23) and 9.4% (36)). The highest proportion of nodules that did not fit into the three primary categories is 18.5% (32), greater than those computed by other authors (23, 30), while other studies reported minimal or no additional nodules.

#### 3.5.2 Nodule margins and localization

Beyond internal tissue composition and attenuation, the external contour and anatomical distribution of a nodule provide additional, independent diagnostic clues. Evaluating nodule margins for irregularities or spiculation, combined with identifying their specific lobar localization, further refines the qualitative risk profile established by the nodule’s density.

**Figure 8:**
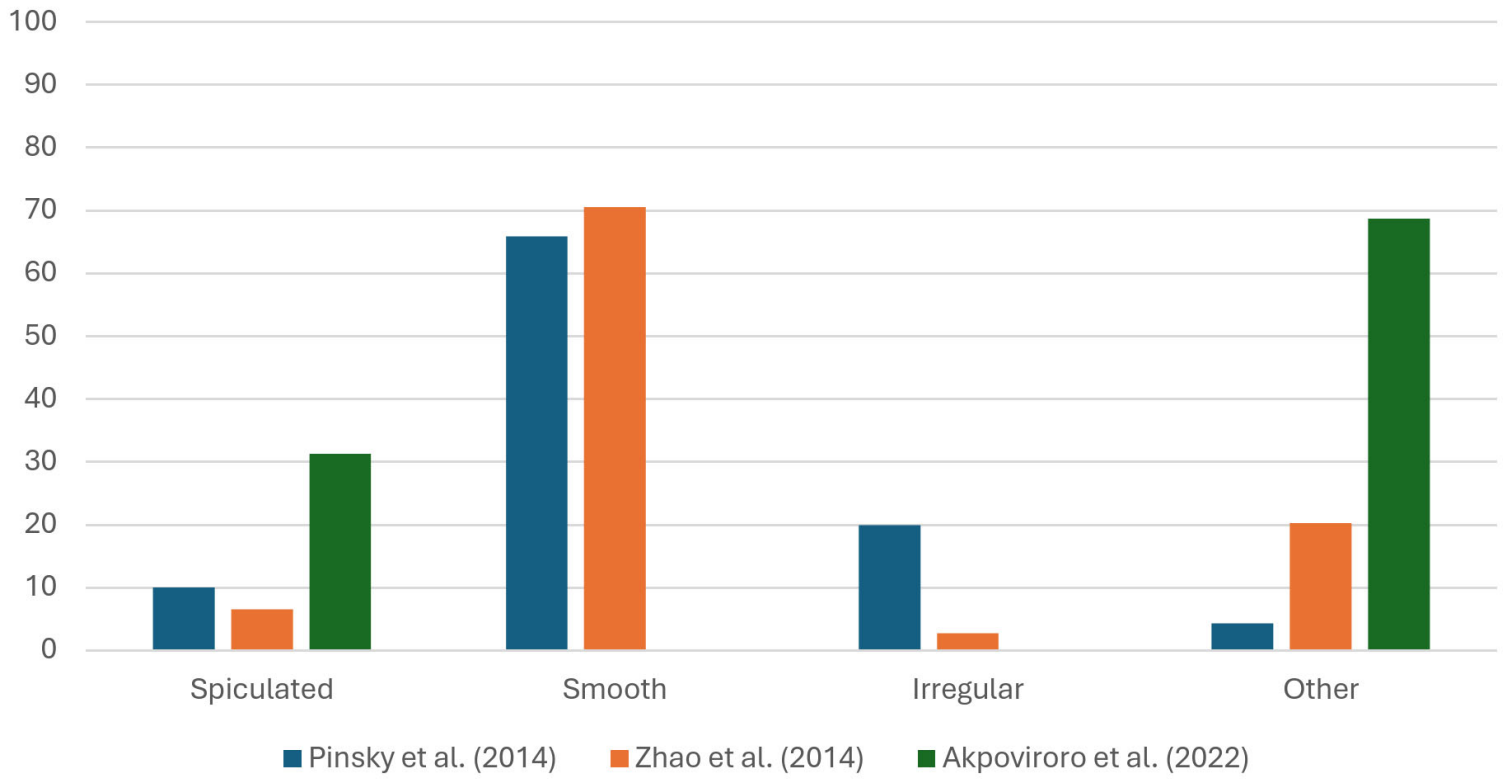
Distribution of pulmonary nodules based on three margin categories: “Spiculated,” “Smooth,” “Irregular,” “Other”. These classifications were not consistently present across all studies (23, 24, 44). In Akpoviroro et al. (2022) (44), nodule margins were categorized as spiculated vs. non-spiculated, with non-spiculated nodules grouped under “Other” for consistency. Similarly, Pinsky et al. (2014) (23) referred to “Poorly Defined” margins, which were considered equivalent to the “Irregular” category.

**Figure 8** summarizes nodule margin distribution across studies, standardized into three categories: spiculated, smooth, and irregular (23, 24, 44), despite variations in original classifications (e.g., spiculated vs. non-spiculated, or “poorly defined” margins grouped as irregular). Smooth margins are predominant (65.9% and 70.54%) (23, 24), whereas spiculated nodules are generally less frequent (~10%) (23, 24)) but reach higher proportions in some datasets (31.3%) (26). Irregular margins show variable prevalence (19.9% vs. 2.7%) (23, 24), and are absent in some studies (26). The “Other” category varies widely, from minimal (4.3%) (23) to dominant (68.7%) (44), reflecting heterogeneity in classification approaches.

**Figure 9:**
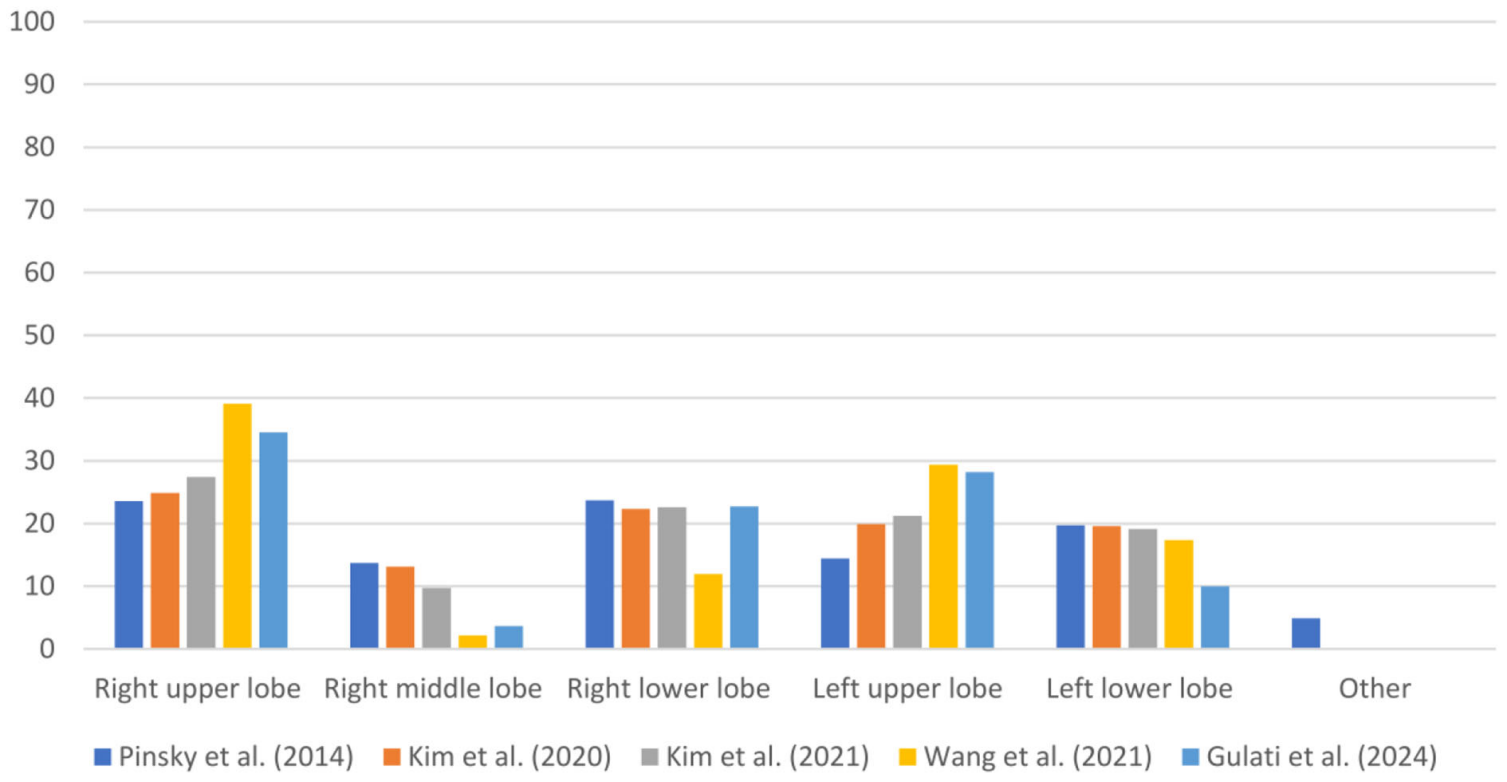
Distribution of pulmonary nodules by location (lung lobes) Across Multiple Studies. To represent the distribution of nodules within the lungs, five primary categories (right upper lobe, right middle lobe, right lower lobe, left upper lobe, left lower lobe and other) were selected and expressed as percentages. These categories were consistently reported across all authors. However, Pinsky et al. (2014) (23) and Gulati et al. (2024) (38) included additional classifications, such as Lingula and Trachea or Main Bronchus. To maintain consistency in comparison, these additional categories were reclassified under the “Other” category.

**Figure 9** presents nodule distribution by anatomical location across studies (23, 30–32, 38), with additional sites (e.g., lingula, trachea) grouped as “Other” for consistency. The right upper lobe is the most frequent location (23.6–39.13%) (23, 30–32, 38), followed by the left upper lobe (14.4–29.35%) (23, 30, 31, 38). The right and left lower lobes show relatively consistent distributions (≈20–25%), although lower values are occasionally reported (11.96% and 10%) (25, 41). The right middle lobe consistently has the lowest prevalence (≈2–15%). Other locations are infrequent and inconsistently reported (23, 38).

### 3.6 Disease staging at diagnosis

**Figure 10:**
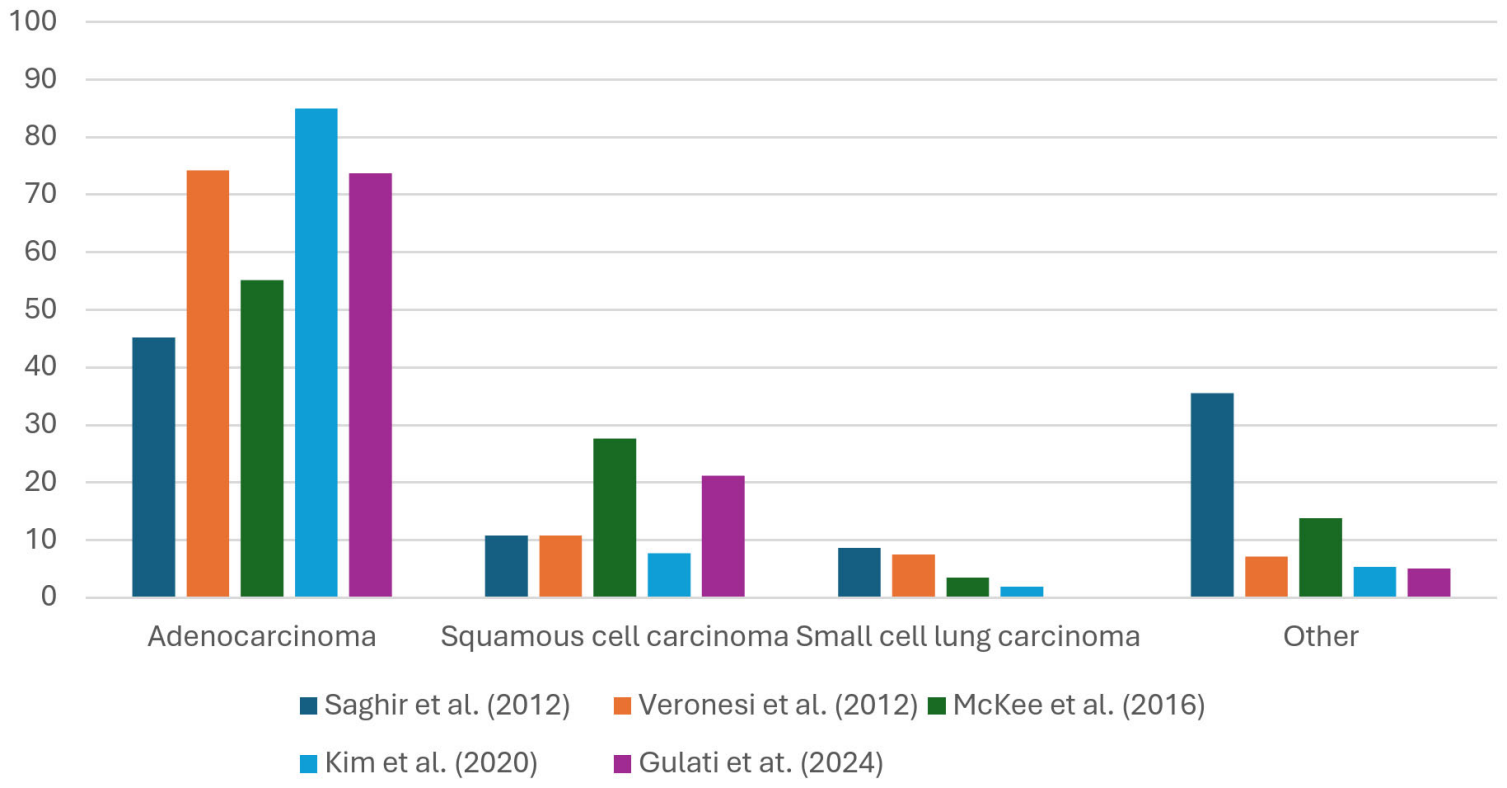
Distribution of LC histological types. Three primary histopathological LC types (adenocarcinoma, squamous cell carcinoma, small cell lung carcinoma) were reported uniformly by the studies on suspicious LC nodules, while rarer subtypes were grouped under the “Other” category.

**Figure 10** summarizes the distribution of major histopathological types across five studies (14, 18, 25, 30, 38), grouping less frequent subtypes as “Other.” Adenocarcinoma is the predominant type, ranging from 45.17% (14) to 85% (30), with most studies reporting ~75% (18, 38). Squamous cell carcinoma shows greater variability, from 7.7% (30) to 27.59% (38), with intermediate values around 10–21% (14, 18, 25). Small cell lung carcinoma remains consistently low (<10%), with values between 1.9% and 8.6% (14, 18, 25, 30). The “Other” category varies widely, reaching 35.49% in one study (14) and remaining lower in others (5–13.79%) (25, 28, 30), reflecting differences in classification breadth.

**Figure 11:**
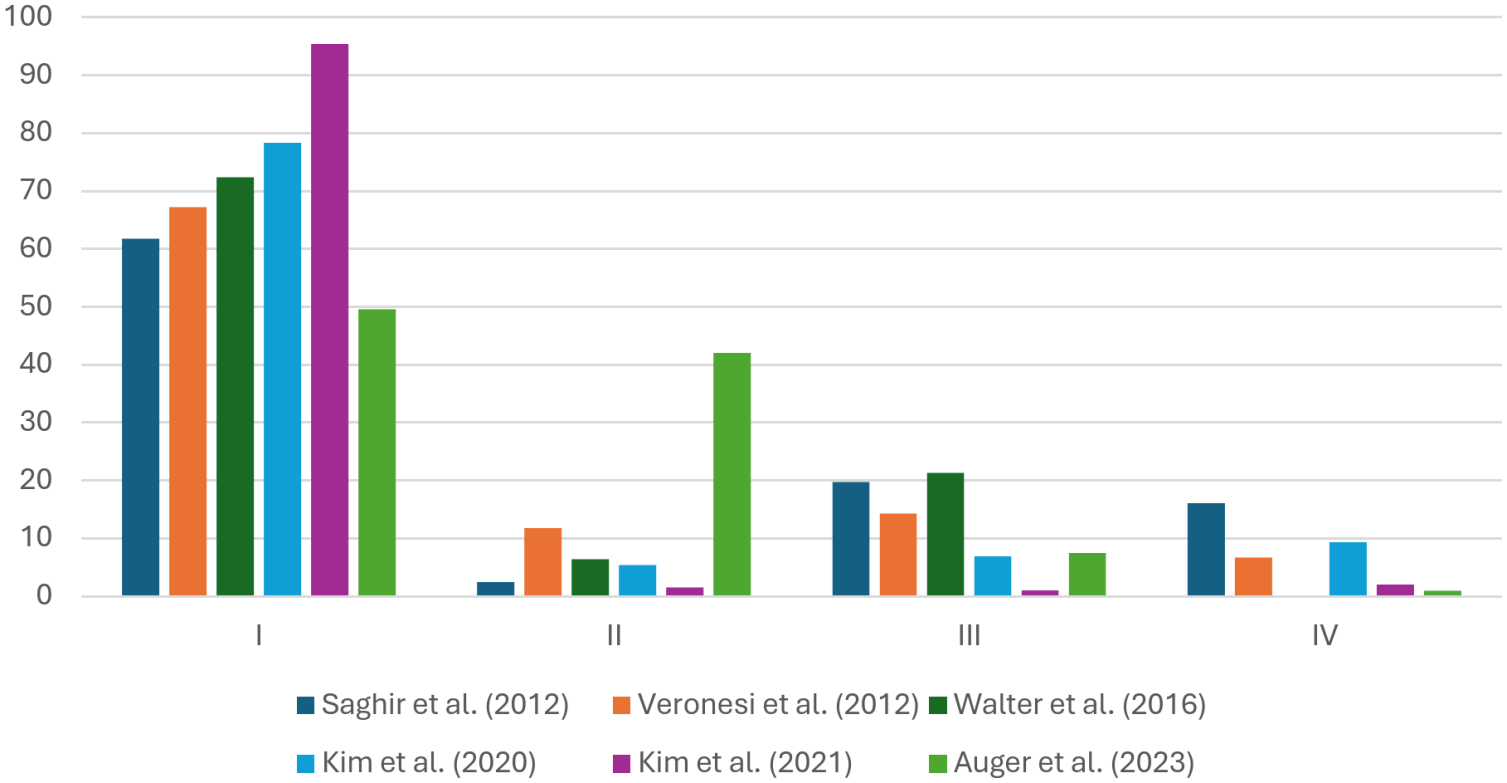
Distribution of LC nodules across AJCC stages. The nodules were classified according to the AJCC 9th edition, grouped into the four main stages (I-IV) by consolidating the subgroups **(A–C)** within their respective primary stages, and were graphically represented as percentages.

**Figure 11** presents stage distribution consolidated into four categories (I–IV, AJCC 9th edition) for comparability across six studies (14, 18, 26, 30, 31, 35). Stage I predominates in all cohorts, ranging from 49.53% (35) to 95.41% (31), with most studies reporting 67.2–78.33% (18, 26, 30). Stage II is generally infrequent (<12%), except in one study (42.06%) (35). Stage III shows moderate variability (7.48–21.28%) (14, 18, 26, 35), while remaining low (<7%) in other cohorts (30, 31). Stage IV is the least frequent overall but reaches 16.05% in one study (14), with lower values reported elsewhere (0–9.36%) (18, 26, 30, 35).

## 4 Discussion

We were the first authors to conduct a comparative analysis of nodules based on VDT. Although each study reported VDT according to its own specific criteria, we consolidated these characteristics and reclassified them to ensure comparability across datasets.

### 4.1 VDT as a marker of pathological lung nodules

Pulmonary nodule distribution varies heavily according to VDT. To eliminate ambiguity and maintain consistent terminology throughout this review, we apply the established clinical categorizations: fast-growing (<400 days), slow-growing (400–600 days), and indolent (>600 days) (18, 22). Fast-growing nodules severely predominate among malignant findings, indicating highly aggressive tumors. In contrast, indolent nodules (>600 days) frequently represent benign lesions or overdiagnosed cases, highlighting the heterogeneity in tumor behavior and the absolute necessity of applying standardized VDT thresholds in clinical practice.

From a diagnostic perspective, VDT shows distinct strengths and limitations. It is highly effective for ruling out malignancy (high NPV), ensuring reliable identification of benign nodules. However, its ability to confirm malignancy is limited by low PPV, resulting in a substantial rate of false positives and potential overtreatment. Although nodules with shorter VDTs are more frequently classified as malignant, PPV does not increase proportionally with malignancy risk, indicating limited discriminatory power. Consequently, VDT should not be used as a standalone marker but integrated with complementary diagnostic tools, such as PET-CT or biopsy, to improve accuracy and reduce unnecessary interventions.

### 4.2 Smoking – the main LC risk factor and VDT

Given that VDT has limitations as an isolated diagnostic marker, it is crucial to evaluate how patient-specific clinical factors influence nodule growth kinetics, with smoking status being the most prominent. Nodule growth dynamics differ meaningfully by smoking status. Never-smokers exhibit a lower median VDT, indicating that their nodules generally grow at a faster rate compared to those of ever-smokers. This may reflect distinct underlying tumor biology or etiologies in this population, such as a greater likelihood of aggressive molecular subtypes or non-smoking-related carcinogenic pathways.

However, ever-smokers demonstrate greater variability in nodule growth. At the same time, the greater variability in VDT among ever-smokers’ points to a heterogeneous risk profile within this group, likely influenced by cumulative exposure to tobacco and its interaction with other risk modifiers (e.g., age, genetic susceptibility, comorbidities). This hypothesis is supported by recent literature investigating the biological divergence of lung cancers in different populations. For instance, an eight-year observational study by Kakinuma et al. corroborates our findings, demonstrating that screen-detected lung cancers in never-smokers exhibited significantly faster growth kinetics (median VDT of 1,130 days) compared to those in smokers (median VDT of 6.6 years) (45). This distinct trajectory highlights that ground-glass and subsolid nodules, which are highly prevalent in never-smokers (particularly in Asian populations), represent a different disease entity with unique genetic insights and growth patterns compared to the smoking-induced solid tumors typically found in ever-smokers (31, 45).

This variability may complicate risk stratification in ever-smokers, which points to the importance of integrated models that consider multiple factors beyond VDT alone. The large standard deviation across all groups highlights significant inter-individual differences, reinforcing the idea that VDT lacks discriminatory power as a standalone marker for malignancy risk and is insufficient for comprehensive risk assessment.

The observed variability in VDT between never-smokers and ever-smokers underscores the clinical necessity of factoring patient history into nodule evaluation. Because never-smokers tend to exhibit faster-growing nodules compared to the highly heterogeneous growth rates seen in ever-smokers, screening algorithms must adapt their VDT risk thresholds based on smoking status rather than applying a universal baseline, ensuring more tailored and effective clinical interventions.

### 4.3 The need for an integrative and predictive VDT-based model

The substantial variability in VDT across different patient profiles, such as smoking history, underscores the critical need to transition from single-variable assessments to comprehensive, integrative predictive models. Histopathological subtypes such as LCLC, SCLC, and NSCLC/SCLC exhibit the shortest VDTs (~100 days), reflecting high proliferative activity and aggressive behavior (26). In contrast, nodules with VDT values closer to 200 days still demonstrate rapid growth but may indicate slightly less aggressive disease. Notably, all malignant nodules in the analyzed cohort had VDT <200 days, underscoring the clinical relevance of growth kinetics for malignancy assessment and the need for prompt diagnostic evaluation. The analysis of median VDT across pathological stages reveals significant differences in tumor growth dynamics, with the distribution of new, fast-growing, and indolent nodules varying considerably according to disease stage.

Early-stage LC (Stages I and II) exhibits a wider VDT range and a median exceeding 200 days, suggesting greater variability in tumor proliferation rates. Some of these tumors may be indolent or slow-growing, while others may have the potential to progress more rapidly. Stage I exhibits a high proportion of both indolent and fast-growing nodules, reflecting the heterogeneity of early-stage LC. In Stage II, there is a notable decline in detected nodules, likely due to early interventions or a transitional phase before disease progression. This is related to a high degree of biological variability in early-stage tumors.

Advanced-stage lung cancer (Stages III–IV) is characterized by shorter VDTs (median 65 and 128 days, respectively) and reduced variability, reflecting accelerated tumor proliferation. Stage III is associated with an increase in new nodules, suggesting dissemination, whereas Stage IV is dominated by fast-growing lesions, indicating advanced progression and metastatic consolidation. These patterns support a direct relationship between tumor stage and growth rate. The prognostic value of integrating VDT into predictive models was recently reinforced by Tang et al., who investigated the stage shift growth of early-stage lung adenocarcinomas presenting as subsolid nodules. Their study demonstrated that a multidimensional nomogram incorporating VDT, alongside clinical characteristics and qualitative CT semantic features, yielded a significantly higher predictive performance for stage shift (AUC = 0.877) compared to models relying solely on clinical and radiological features (39). Furthermore, their analysis identified that a VDT of 400 days serves as a crucial, independent predictor of aggressive growth behavior and rapid stage progression. These findings emphasize that integrating VDT into comprehensive risk models is highly effective for informing targeted follow-up protocols and guiding surgical timing.

Despite this, lung cancer is often diagnosed at metastatic stages, with a 5-year survival of 10–15% (9, 10). In contrast, early-stage disease has markedly better outcomes (>80% in Stage I vs. near 0% in Stage IV) (11, 12), although variability across cohorts remains (10). Screening programs are therefore essential, as early-stage disease is frequently asymptomatic. The NELSON trial demonstrated that LDCT enables earlier detection, with 59.6% of cases diagnosed at Stage IA–IB and only 9.4% at Stage IV (4), supporting its effectiveness (2, 14). However, screening also carries risks, including overdiagnosis, radiation exposure, psychological burden, and false-positive-related morbidity (33, 43, 46).

LDCT screening reduces follow-up imaging and invasive procedures compared to chest X-ray (by 25–37%) (37), highlighting its clinical utility. The observed variability in VDT further supports a personalized approach to nodule assessment. Integrating VDT with clinical, radiological, and biomarker data can improve risk stratification and guide management, enabling conservative follow-up for slow-growing nodules and expedited evaluation for rapidly growing lesions.

The value of more comprehensive research on estimated nodule growth and VDT is supported by favorable results from a case-control study in which the predicted VDT of patients with T1-stage lung adenocarcinoma was assessed using an AI-powered diagnostic system (47). Although further investigation is needed to validate the use of digital tools for assessing predicted VDT and distinguishing between indolent and fast-growing nodules, the perspective of integrating such tools into LCS is promising. Furthermore, the variation in VDT distributions across datasets - particularly at the high end - suggests underlying heterogeneity in patient populations, screening protocols, and classification criteria. This highlights the critical importance of standardizing VDT measurement and reporting to ensure consistency and comparability across studies. So, these findings support the incorporation of VDT thresholds into risk stratification models and patient management algorithms, aiming to improve early detection, reduce unnecessary interventions, and guide more effective treatment planning.

VDT variability strongly correlates with both histopathological aggressiveness and disease stage, reinforcing its value as a prognostic indicator. However, the considerable inter-individual differences emphasize the clinical imperative to transition from isolated VDT assessments toward integrative, multidimensional models. Incorporating VDT alongside clinical history, nodule morphology, and automated digital predictive tools will ultimately refine risk stratification and allow for truly personalized lung cancer screening pathways.

### 4.4 Beyond CT-only-based VDT

While integrative models leveraging CT-derived VDT and clinical history are foundational, further diagnostic accuracy can be achieved by incorporating functional metabolic imaging. In the analyzed combined cohort, patients were classified based on disease stage and PET-CT results. Stage I was primarily composed of slow-growing positive nodules and other negative nodules, while other positive nodules accounted for 48%, indicating metabolic activity even in early-stage disease. Stage II showed a sharp decline in slow-growing nodules, likely due to disease progression or treatment response, with other positive nodules (16%) suggesting a transition toward more aggressive tumors. Stage III was dominated by other positive nodules, with no slow-growing positive or other negative nodules present, emphasizing the shift toward higher metabolic activity. Stage IV exhibited fewer total nodules, with only PET-CT positive nodules remaining, characteristic of advanced-stage disease with increased metabolic activity and potentially more aggressive tumor progression.

The correlation between PET-CT results and VDT across pathological stages suggests a correlation between PET-CT positivity and VDT, indicating that lung cancers that are PET-CT positive tend to have a shorter VDT than PET-CT negative tumors within the same. This supports the hypothesis that PET-CT positivity is associated with more aggressive tumor growth.

A notable limitation of this review is the exclusion of a comparative analysis based on Lung-RADS. As previously noted, this exclusion was methodologically necessary due to inconsistent reporting practices across the included studies, where distinct Lung-RADS categories were frequently grouped together. However, this omission impacts the overall analytic completeness of our findings. Lung-RADS is a cornerstone of standardized nodule management that has been proven to substantially reduce false-positive rates and improve the positive predictive value of screening programs (23, 25). Consequently, our inability to harmonize and graphically represent Lung-RADS data limits our capacity to fully evaluate how continuous quantitative measures, such as volume doubling time (VDT), directly contrast with or complement this widely adopted categorical framework.

The correlation between PET-CT positivity and shorter VDT highlights the clinical importance of combining functional metabolic imaging with anatomical growth kinetics. This multimodal approach can significantly enhance the identification of aggressive tumor phenotypes, compensating for the limitations of assessing VDT or morphological features alone, particularly in advanced disease stages.

### 4.5 Other nodules medical imaging features and LCS

In addition to functional imaging and dynamic growth metrics, the fundamental qualitative and morphological features of the nodule on baseline CT must also be systematically evaluated. Nodule size distribution varies across studies, reflecting differences in cohort selection and follow-up criteria. This heterogeneity highlights the need for standardized screening protocols to reduce overdiagnosis and optimize management.

CT features, particularly size and composition, remain central in guiding clinical decisions between routine surveillance, advanced functional imaging (PET/CT), or immediate invasive biopsy (48). The clinical implications of combining these imaging findings are profound: while stable, smooth-margined, and indolent nodules (VDT >600 days) can safely be managed with continued low-dose CT surveillance, large or fast-growing nodules (VDT <400 days) exhibiting high-risk qualitative features (e.g., spiculated margins or part-solid attenuation) necessitate immediate diagnostic workup. Bridging continuous volumetric growth data with these distinct qualitative phenotypes allows clinicians to safely minimize false-positive biopsies while aggressively targeting true malignancies (18, 22). If interval growth is defined by three-dimensional VDT rather than one-dimensional diameter thresholds, estimations of nodule progression are significantly more precise.

If interval growth is defined by three-dimensional VDT or one-dimensional diameter thresholds, nodule progression can vary greatly. VDT measures volumetric growth kinetics continuously, while linear assessments use fixed size-increase thresholds that can alter tumor progression. When studying the natural history of subsolid nodules, Tang et al. (2019) defined ‘true growth’ as a diameter increase of ≥ 2 mm and’substantial growth’ as ≥ 5 mm. Their diameter-based thresholds showed that GGNs took 7 years to show true growth and 9 years for substantial growth, while PSNs took 3 years to meet both thresholds (29). This shows that linear diameter thresholds for indolent lesions like GGNs require long-term surveillance (7–9 years), while VDT may detect volumetric neoplastic changes earlier in the clinical course.”

Regarding attenuation, most nodules are classified as solid or part-solid, although methodological differences limit direct comparison across studies (23, 30–32, 36). GGNs remain consistently represented (9–33%) and are clinically relevant due to their malignant potential despite often indolent behavior (49).

Margin analysis shows that smooth nodules predominate, while spiculated and irregular margins - more suggestive of malignancy - are less frequent. Variability across studies, including broader “Other” categories (44), reflects differences in classification approaches.

Nodule localization is inconsistently reported but may have clinical relevance. Available data indicate a predominance in the right upper lobe, followed by the lower lobes and left upper lobe (12–30%), with the right middle lobe least affected (2–14%) (23, 30–32, 38). Standardizing anatomical reporting could improve risk stratification, potentially supported by digital tools for detection and mapping.

Histopathologically, adenocarcinoma is the most frequent subtype (>40%), followed by squamous cell carcinoma (~30%) and small cell carcinoma (<10%), consistent with global trends. Variability in “Other” categories reflects differences in cohort composition. Persistent subsolid nodules are strongly associated with adenocarcinoma and represent a distinct biological entity (50).

Morphological features such as nodule diameter, attenuation, and margins exhibit significant variability across screening cohorts, complicating standardized management. The clinical implication is that these qualitative and semi-quantitative features must be systematically integrated with dynamic markers like VDT into comprehensive, evidence-based protocols to accurately differentiate benign from malignant lesions and effectively minimize the harms of overdiagnosis and unnecessary invasive procedures.

Here is the transcribed text with the requested citation format integrated, perfectly mapping the methodological findings to the specific studies you included in your QUADAS-3 analysis:

### 4.6 Impact of study quality and risk of bias on review findings

The reliability of this review’s findings must be interpreted in the context of the methodological quality of the included literature. Based on our formal QUADAS-3 assessment, the evaluated studies demonstrated considerable strengths, particularly regarding the index test and statistical analysis. Almost all studies (96%) had a low risk of bias in the index test domain, utilizing objective, pre-specified criteria (such as Lung-RADS or volumetric thresholds) without knowledge of the final diagnosis (22, 25, 26). Additionally, 80% of the studies employed robust statistical analyses that appropriately handled missing data and clustered nodule tracking.

However, 60% (15 out of 25) of the included studies exhibited a high overall risk of bias, predominantly driven by challenges inherent to radiological screening research. Specifically, 36% of the studies had a high risk of bias regarding participant selection, often due to the use of retrospective, multi-gate (case-control) designs, or the exclusive inclusion of surgically resected nodules (15, 27, 29, 32, 34–36, 38, 39). Such highly restricted cohorts may overestimate disease prevalence and do not perfectly represent an unselected, asymptomatic screening population.

This inherent participant selection bias within the primary literature consequently translates into a broader selection bias for our review as a whole. By intentionally prioritizing studies with complete and extractable quantitative data, our synthesis is disproportionately influenced by retrospective cohorts of surgically resected or highly suspicious nodules, which are inherently more likely to have detailed pathological and imaging data recorded. Therefore, the nodule characteristics, growth kinetics, and malignancy rates discussed in this review may reflect a more aggressive subset of nodules than those typically encountered in a general, unselected LCS program. Readers should interpret the aggregated findings with caution, recognizing that the strict criteria necessary to perform this comparative analysis inherently skew the pooled data toward higher-risk clinical scenarios.

Furthermore, 24% of the studies suffered from differential or partial verification bias regarding the target condition (25, 30, 35, 37, 42, 44). Because it is clinically and ethically unjustifiable to biopsy all screen-detected nodules, different reference standards were frequently applied (e.g., histopathology for highly suspicious nodules versus long-term radiological surveillance for low-risk nodules) (31, 44).

While these methodological limitations and applicability concerns (such as the inclusion of never-smokers in some Asian cohorts) slightly temper the generalizability of certain specific numerical accuracy estimates (30, 31)], the overall strong adherence to standardized index testing and rigorous long-term follow-up within the selected studies mitigates major concerns. Consequently, VDT thresholds and morphological risk stratification strategies synthesized in this review provide a highly robust, evidence-based foundation for clinical management, though future large-scale, prospective, single-gate studies remain necessary to perfectly validate these findings in unselected populations (1, 18, 22, 39).

### 4.7 Heterogeneity in imaging protocols, segmentation, and patient populations

A critical appraisal of the included literature reveals that conflicting findings across studies are frequently driven by profound methodological and demographic heterogeneity. First, the definition of ‘fast-growing’ or ‘high-risk’ VDT varies considerably; while some protocols utilize a strict 400-day threshold to indicate malignancy (21, 22), others demonstrate that extending the VDT cutoff to 590 or 600 days provides optimal sensitivity and negative predictive value for identifying lung cancer in new solid nodules (1).

Furthermore, significant heterogeneity exists in imaging protocols and segmentation differences. The studies evaluated utilized a wide array of CT hardware, ranging from 16-slice to 256-slice multidetector scanners, with slice thicknesses varying from 1.0 mm to 2.5 mm (21, 30, 51). More importantly, the method of nodule measurement plays a critical role in VDT accuracy. Studies utilizing semi-automated 3D volumetric software (such as LungCARE) demonstrate high reproducibility and reliability (21, 26). Conversely, VDT calculations based on manual 2D diameter measurements are significantly less precise and suffer from higher inter-reader variability, which can artificially alter perceived growth rates and overestimate nodule size (22). Therefore, the diagnostic accuracy and false-positive rates of diameter-based VDT studies cannot be perfectly equated with those utilizing volumetric segmentation.

Finally, conflicting clinical outcomes can often be explained by the underlying patient populations. For example, while studies from the NELSON and I-ELCAP trials found that newly detected nodules generally have a higher probability of being malignant compared to baseline nodules (26, 31), research by Kim et al. reported the exact opposite, finding a *lower* cancer probability in new subsolid nodules (31). This conflict is likely attributable to racial and demographic differences; the latter study evaluated an Asian population with a high proportion of never-smokers (nearly 50%), a demographic known to have a distinct disease entity characterized by a higher prevalence of indolent subsolid nodules compared to the Western heavy-smoker cohorts typically evaluated in landmark screening trials (30, 31).

### 4.8 Beyond LCS – acting based on nodules indicative features

Ultimately, the synthesis of these dynamic, functional, and morphological features is essential to guide the complex clinical management and treatment decisions following a positive screening result. Most cases are diagnosed at Stage I, particularly in recent studies, reflecting improved screening performance, whereas earlier studies report higher proportions of advanced stages (III–IV). This trend underscores the effectiveness of screening in enabling early detection, although further optimization is needed to minimize late-stage diagnoses and associated mortality.

Clinical management of pulmonary nodules remains complex and largely dependent on judgment (52), with evidence of unnecessary invasive procedures in patients with benign nodules (2). Nodule characteristics such as size, attenuation, and margins are therefore critical for risk stratification and decision-making (23).

Newly detected nodules are often benign but may carry a higher malignancy risk compared to baseline nodules, although data on their initial characteristics and progression remain limited (26). Importantly, high-risk individuals may still develop lung cancer after negative screening, highlighting the need for continued surveillance (27).

Integrating LDCT findings with emerging biomarkers may further improve early detection and risk stratification, representing a promising direction for advancing lung cancer screening (15, 34).

Digital tools, particularly artificial intelligence (AI) and machine learning, are increasingly valuable for refining nodule surveillance, especially when focused on quantitative metrics like VDT. Rather than relying solely on manual diameter measurements, deep learning models and radiomic feature extraction can automate three-dimensional volume segmentations, thereby significantly improving the accuracy, speed, and reproducibility of VDT calculations.

Evidence-based conclusions drawn from recent literature demonstrate the high quantitative accuracy of these models. A systematic review of 43 lung cancer risk prediction models revealed that deep learning algorithms consistently outperform traditional statistical methods, with the majority of deep learning models achieving an area under the curve (AUC) greater than 0.80, and some reaching as high as 0.970 for predicting malignancy (12). Similarly, a proof-of-concept radiomics biomarker utilizing a random survival forest method demonstrated excellent time-dependent predictive accuracy for lung cancer diagnosis within 12 months, achieving an AUC of 0.928 in the training set and 0.888 in the testing set, alongside a time-dependent sensitivity of 0.968 and a specificity of 0.975 (32). These metrics provide robust evidence that radiomic features can effectively differentiate between indolent lesions and fast-growing malignant nodules.

Furthermore, by integrating automated VDT growth metrics with intrinsic radiomic features, AI tools can create comprehensive, multidimensional risk models that more effectively differentiate between indolent lesions and fast-growing malignant nodules. This targeted application of digital tools minimizes inter-reader variability and directly maximizes the prognostic value of VDT-centered analysis.

Despite the promising potential of AI and digital tools, their integration into routine clinical workflows faces significant feasibility and implementation challenges. First, many deep learning algorithms and radiomic models are still in early developmental stages and lack extensive external validation in large, prospective clinical trials, making their generalized clinical application premature (12, 53).

However, despite these promising quantitative metrics, their integration into routine clinical workflows faces significant implementation challenges, primarily regarding data generalizability. A critical limitation is the lack of external validation across multicenter datasets. Among the extensively developed lung cancer radiomics models, only 23% have been externally validated, with the vast majority relying on single-center or highly homogenous retrospective cohorts (12).

This barrier is not unique to lung cancer screening, but represents a systemic hurdle in the broader landscape of medical AI. For instance, a recent scoping review of AI models for Alzheimer’s disease digital biomarkers found that while models achieved a high average AUC of 0.887 for disease classification, only 2 out of 86 studies incorporated external validation, severely limiting their clinical reliability (54). To overcome these generalized limitations, future lung cancer radiomics research must adopt robust, multi-center validation frameworks similar to those recently demonstrated in other advanced medical imaging domains. For example, recent developments in weakly supervised AI for automated polyp segmentation have successfully utilized diverse, multi-center datasets (such as PolypGen) to rigorously test algorithmic robustness across varying clinical environments, hardware types, and lighting conditions, achieving high accuracy (e.g., mean Dice coefficient of 88.27%) precisely because the models were forced to generalize beyond localized training data (55).

Therefore, it is crucial to distinguish between evidence-based conclusions and speculative future directions regarding AI in nodule management. Based on current evidence, radiomics and AI can reliably serve as powerful *assistive* tools to automate laborious segmentation tasks, reduce inter-reader variability, and provide secondary risk stratification metrics (such as precise VDT tracking) (12, 32). However, the concept of utilizing AI algorithms as autonomous, primary decision-makers that independently dictate patient management pathways remains a speculative future direction. Until these models undergo rigorous, prospective validation in large-scale, multi-center clinical trials to prove their safety and generalizability across diverse demographic and technological landscapes, clinical judgment and established multidisciplinary protocols must continue to guide the management of suspicious pulmonary nodules.

Deploying these advanced technologies requires substantial institutional investment in both equipment and information technology infrastructure. Establishing systems like integrated electronic health records with natural language processing capabilities or specialized tracking registries is often resource-intensive and may not be readily available in all community healthcare settings (56). Finally, successful implementation goes beyond the technology itself; it demands dedicated personnel, including trained lung nodule coordinators and multidisciplinary teams, to manage the complex screening workflow and ensure appropriate follow-up (51, 53). Consequently, the transition of these digital tools from conceptual models to standardized clinical practice requires overcoming considerable financial, infrastructural, and validation barriers.

Given these substantial variances in imaging hardware, segmentation techniques, VDT mathematical thresholds, and baseline patient risk profiles, readers must exercise caution to avoid overgeneralizing the findings of this review. The aggregated data synthesis presented herein is derived from a limited dataset of highly curated studies. Because many of these studies applied differing reference standards and highly specific inclusion criteria (e.g., exclusively analyzing surgically resected adenocarcinomas), the summarized growth kinetics and malignancy probabilities may not universally apply to all demographic groups or all community-based lung cancer screening programs (15). Standardized, multi-center, prospective validations are required to harmonize these fragmented criteria into a universally applicable management protocol.

To further overcome the limitations of conventional screening modalities, the future of nodule management lies in the integration of novel molecular mechanisms and advanced imaging nanotechnologies. First, advances in molecular imaging are rapidly evolving to improve the early detection of lung cancer, offering higher specificity than traditional metabolic imaging and allowing for the precise visualization of molecular alterations at the cellular level (57). Coupling these advanced imaging techniques with next-generation therapeutic vehicles, such as tumor cell-targeting and tumor microenvironment-responsive nanoplatforms, holds tremendous clinical promise. As demonstrated in recent oncological models, these multimodal nanoplatforms can be engineered to react to the specific tumor microenvironment (e.g., hypoxia or slight acidity) to provide highly targeted, imaging-guided photodynamic and chemodynamic therapies, thereby minimizing systemic side effects while maximizing local tumor ablation (58). Translating these imaging-guided nanoplatforms to pulmonary medicine could revolutionize the localized treatment of early-stage, screen-detected lung cancers.

In parallel, understanding the epigenetic regulators driven by environmental risk factors is essential for refining risk stratification models for patients with indeterminate nodules. While smoking remains the primary risk factor, environmental pollutants significantly alter tumor biology. Recent evidence highlights the critical role of PIWI-interacting RNAs (piRNAs) in environmentally induced malignancies. For instance, long-term exposure to fine particulate matter (PM2.5) promotes lung cancer progression through the significant up-regulation of the specific piRNA PMLCPIR. This piRNA attenuates nucleolin (NCL) binding, which in turn enhances ITGB1 expression and activates the oncogenic PI3K/AKT signaling pathway (59). Integrating these novel epigenetic biomarkers into lung cancer screening algorithms could significantly enhance the identification of high-risk individuals—especially those with heavy environmental pollutant exposures—and guide the development of precision interventions.

### 4.9 Study limitations and generalizability

This review is subject to several methodological and analytical limitations that must be carefully considered when interpreting its findings. First, the review is limited by inherent selection bias. Enforcing strict data-completeness criteria for inclusion inherently favored retrospective studies and cohorts of patients who underwent surgical resection or invasive biopsies (15, 36). Consequently, the synthesized data overrepresents highly suspicious and malignant nodules, overestimating the overall malignancy risk and presenting a cohort that does not perfectly mirror an unselected, asymptomatic screening population.

Second, the restriction of our literature search to English-language publications introduces language bias. This exclusion may omit critical findings, localized clinical guidelines, and diverse screening program outcomes published in other languages, potentially neglecting relevant epidemiological differences observed in non-Western populations (45, 60).

Third, the lack of a formal meta-analysis limits the quantitative strength of our conclusions. As a narrative review, we synthesized qualitative and quantitative findings but did not perform statistical pooling of diagnostic performance metrics or hazard ratios. Therefore, while the synthesized VDT thresholds and malignancy rates highlight important clinical trends, they do not provide definitive, statistically weighted cut-offs.

Fourth, there is substantial variability in imaging protocols across the included studies. The diagnostic accuracy of nodule characterization is heavily influenced by differences in CT hardware (ranging from 16- to 256-slice scanners), slice thicknesses (ranging from 1.0 mm to 2.5 mm), and radiation dose settings (21, 39, 51). Furthermore, discrepancies between manual 2D diameter measurements and semi-automated 3D volumetry significantly impact the reproducibility and precision of VDT calculations, complicating the direct comparison of growth kinetics across different trials (22).

Explicitly, these compounding limitations restrict the generalizability of our findings. The aggregated growth kinetics, optimal VDT thresholds, and malignancy probabilities derived from this highly curated and methodologically heterogeneous dataset reflect a higher-risk subset of patients and may not universally apply to all demographic groups or standard, community-based lung cancer screening programs. Clinicians should exercise caution when extrapolating these narrative findings to routine practice, and future large-scale, prospective, and multicenter studies are required to establish universally applicable nodule management guidelines.

## 5 Conclusion

This study highlights the clinical value of VDT as a marker of tumor aggressiveness and growth kinetics in lung cancer. All malignant nodules in the analyzed cohort demonstrated a VDT below 200 days, with particularly short VDTs observed in aggressive histopathological subtypes such as LCLC, SCLC, and combined NSCLC/SCLC. These findings emphasize the importance of VDT as a tool for distinguishing fast-growing malignancies requiring prompt intervention. VDT also varied across pathological stages, reflecting differences in tumor behavior from early to advanced disease. While rapidly proliferating tumors with narrow VDT ranges dominated advanced stages, early-stage lung cancers showed wider variability, including both indolent and fast-growing nodules. The strong observational correlation between PET-CT positivity and shorter VDTs across stages, reinforces the association between metabolic activity and aggressive tumor behavior. This progression underlines the need for timely diagnosis and intervention to improve survival outcomes. Additionally, differences in VDT by smoking status - faster-growing nodules in never-smokers and greater variability among ever-smokers - highlight the complex interplay of tumor biology and exposure history, underscoring the need for multifactorial risk assessment models that extend beyond VDT alone.

The integration of VDT into personalized risk assessment models - alongside imaging features, clinical history, and biomarkers - can refine screening and management strategies. Incorporating digital tools such as deep learning technology can further enhance this process by enabling automated, accurate VDT prediction and advanced analysis of imaging data, thereby improving risk stratification. Standardizing VDT measurement and reporting across studies is essential to ensure consistent risk stratification and optimize patient outcomes in lung cancer screening programs.
